# A regulatory network involving Rpo, Gac and Rsm for nitrogen-fixing biofilm formation by *Pseudomonas stutzeri*

**DOI:** 10.1038/s41522-021-00230-7

**Published:** 2021-07-01

**Authors:** Liguo Shang, Yongliang Yan, Yuhua Zhan, Xiubin Ke, Yahui Shao, Yaqun Liu, Hua Yang, Shanshan Wang, Shuling Dai, Jiasi Lu, Ning Yan, Zhimin Yang, Wei Lu, Zhu Liu, Shanchun Chen, Claudine Elmerich, Min Lin

**Affiliations:** 1 Biotechnology Research Institute/Key Laboratory of Agricultural Genomics (MOA), Chinese Academy of Agricultural Sciences, Beijing, China; 2 School of Basic Medicine, Guangxi University of Chinese Medicine, Nanning, China; 3 Key Laboratory of Tropical Biological Resources of Ministry of Education, School of Life and Pharmaceutical Sciences, Hainan University, Haikou, China; 4 Citrus Research Institute, Southwest University/Chinese Academy of Agricultural Sciences, Chongqing, China; 5 Institut Pasteur, Paris, France

**Keywords:** Biofilms, Microbial genetics

## Abstract

Biofilm and nitrogen fixation are two competitive strategies used by many plant-associated bacteria; however, the mechanisms underlying the formation of nitrogen-fixing biofilms remain largely unknown. Here, we examined the roles of multiple signalling systems in the regulation of biofilm formation by root-associated diazotrophic *P. stutzeri* A1501. Physiological analysis, construction of mutant strains and microscale thermophoresis experiments showed that RpoN is a regulatory hub coupling nitrogen fixation and biofilm formation by directly activating the transcription of *pslA*, a major gene involved in the synthesis of the Psl exopolysaccharide component of the biofilm matrix and *nifA*, the transcriptional activator of *nif* gene expression. Genetic complementation studies and determination of the copy number of transcripts by droplet digital PCR confirmed that the regulatory ncRNA RsmZ serves as a signal amplifier to trigger biofilm formation by sequestering the translational repressor protein RsmA away from *pslA* and *sadC* mRNAs, the latter of which encodes a diguanylate cyclase that synthesises c-di-GMP. Moreover, RpoS exerts a braking effect on biofilm formation by transcriptionally downregulating RsmZ expression, while RpoS expression is repressed posttranscriptionally by RsmA. These findings provide mechanistic insights into how the Rpo/Gac/Rsm regulatory networks fine-tune nitrogen-fixing biofilm formation in response to the availability of nutrients.

## Introduction

The term ‘biofilm’ can be defined as a community of microbes adhering to biotic or abiotic surfaces that is protected from environmental stresses by a self-produced extracellular matrix^1,2^. The extracellular matrix, often referred to as extracellular polymeric substances, is composed of exopolysaccharides, proteins and extracellular DNA present in various concentrations depending on the bacterial species^3,4^. The biofilm state provides potential advantages over the planktonic state, including increased resistance to antimicrobial agents, protection from environmental stresses, and improved adaptation to nutrient deprivation^5^. Numerous investigations in recent decades have demonstrated that bacterial biofilm formation is a sequential process governed by complex regulatory networks that differ from one bacterial species to another^1,6^. It is now well accepted that microbial biofilms are the most widely distributed and predominant mode of life on Earth, influencing our lives tremendously in both positive and negative ways^6–9^.

In general, as established in the model bacterium *Pseudomonas aeruginosa*, biofilm development usually begins with attachment to a surface, followed by microcolony formation and production of the extracellular matrix responsible for the biofilm architecture^10–14^. Biofilm formation has been studied intensively in the genus *Pseudomonas*, with an emphasis on genetic elements and molecular mechanisms; Gac/Rsm, c-di-GMP signalling and quorum-sensing (QS) pathways were reported as the main mechanisms leading to biofilm formation^15,16^. The Gac/Rsm signalling pathway involves the GacS/GacA two-component regulatory system, the RNA-binding protein RmsA, and its cognate regulatory non-coding RNAs (ncRNAs)^17,18^. The GacS/GacA two-component system activates the transcription of one or several genes for Rsm ncRNAs, which contain multiple GGA motifs in exposed stem loops of their predicted secondary structures^19^. The GGA motifs allow Rsm ncRNAs to bind the RNA-binding proteins that act as global posttranscriptional repressors, e.g., CsrA (in *Escherichia coli*) and RsmA (in *P. aeruginosa*), controlling important cellular processes, such as secondary metabolism (e.g., metabolism of pyocyanine or the QS signal N-butyryl-homoserine lactone in *P. aeruginosa*), motility, and biofilm formation^17,20^. RsmA specifically recognises and binds to conserved GGA motifs in the 5′-untranslated region (5′-UTR) of target mRNAs, thereby preventing ribosome access and protein translation^17,21^. RsmA controls biofilm formation through direct repression of various target genes, such as *pslA* (involved in the synthesis of the exopolysaccharide Psl) and *sadC* (involved in c-di-GMP synthesis)^22,23^. As a key biofilm regulatory molecule, the second messenger c-di-GMP is synthesised by diguanylate cyclases (DGCs) that bear a GGDEF domain and is degraded by phosphodiesterases (PDEs) that harbour EAL or HD-GYP domains. *P. aeruginosa* encodes several DGCs and PDEs; for example, WspR/SadC/RoeA (DGC) and RocR/BifA (PDE), are absent in the *P. stutzeri* A1501 genome, except for SadC and BifA, which modulate the level of c-di-GMP and influence ‘surface-associated behaviours’ by controlling polysaccharide syntheses^16,24–27^. The *P. aeruginosa* biofilm matrix contains several polysaccharide components, including alginate, pellicle (Pel) and Psl exopolysaccharides^28^. It has been shown that *pslA* is the first gene in the *psl* operon, which comprises 15 cotranscribed genes that are involved in the synthesis of Psl^29^. Although current data relating to the roles of Psl are limited, Psl is a critical component of the *P. aeruginosa* biofilm matrix, which functions as a scaffold, holding biofilm cells together to initiate biofilm development^30^. In addition, evidence demonstrates that biofilm formation is controlled positively by RpoN but negatively by RpoS, suggesting global antagonism between RpoN and RpoS, although there are contradictory reports^31–35^.

Microbial biofilms are common on plant surfaces and have been associated with phytopathogenic infections and colonisation by nitrogen-fixing rhizobacteria^36,37^. Because of dynamically fluctuating conditions in the rhizosphere, the ability of diazotrophic bacteria to form nitrogen-fixing biofilms may confer many ecological advantages and thereby facilitate their physiological and metabolic adaptation to successfully survive in the rhizosphere, a nitrogen-limited environment. An early study compared biofilm formation by a nitrogen-fixing strain of *Klebsiella pneumoniae* with that of two other members of Enterobacteriaceae, *Salmonella enteritidis* and *E. coli*, and showed that the nitrogen-fixing strain formed the densest and most metabolically active biofilms^38^. Many nitrogen-fixing bacteria, such as those of the genera *Rhizobium*, *Gluconacetobacter* and *Azospirillum*, produce biofilms containing various exopolysaccharides^39–42^. For instance, *Sinorhizobium meliloti* produces two symbiosis-promoting exopolysaccharides, succinoglycan and galactoglucan, which function in host specificity and participate in early stages of a host plant infection, biofilm formation, and, most importantly, protection from environmental stresses^43–45^. *Azospirillum* cells are also capable of forming biofilms on both abiotic surfaces and in association with host plants^46^. Previous studies have demonstrated that two response regulator proteins, TyrR and FlcA, were found to be involved in the transcriptional regulation of biofilm formation by *A. brasilense* Sp7 via the production of capsular polysaccharides^42,47^.

The root-associated bacterium *P. stutzeri* A1501 is a rare example of a *Pseudomonas* strain with nitrogen fixation ability^48^. *P. stutzeri* A1501 can survive in the soil, colonise the root surface, and endophytically invade the root tissues of host plants. During evolution, A1501 acquired a nitrogen fixation island with a *nif*-specific regulatory system from a diazotrophic common ancestor^48^. Similar to many other *Pseudomonas* species, the nitrogen regulatory cascade in A1501 comprises the AmtB–GlnK–NtrBC-RpoN global nitrogen regulation proteins and a set of regulatory ncRNAs that control the expression of *nif* genes and the consequent optimal nitrogen fixation in response to nutrient stress^49–52^. Comparative genomics analysis showed that A1501 does not possess the well-known QS systems and does not produce alginate, but it contains genes possibly involved in cellulose biosynthesis and an incomplete *psl* operon^4,48^. It was previously shown that a nonpolar mutation of the *fleQ* gene, encoding FleQ (the main regulator of flagella synthesis), impaired motility and root colonisation but enhanced biofilm formation by *P. stutzeri* A1501^53^. Additionally, Wang et al. investigated the effect of physiological conditions on the formation and architecture of nitrogen-fixing biofilms by *P. stutzeri* A1501^41^. However, the composition of the polysaccharide matrix remains unknown. To date, studies on biofilm formation by nitrogen-fixing rhizobacteria have focused on ecological, physiological and architectural analyses. Despite its importance to microbial adaptation and survival, there is surprisingly little information about the genetics of nitrogen-fixing biofilm formation.

In this work, physiological conditions leading to nitrogen-fixing biofilm formation by the root-associated bacterium *P. stutzeri* A1501 were further investigated. We found that conditions favouring biofilm formation differ between diazotrophic and non-diazotrophic *P. stutzeri* strains, although both strains contain the same set of regulatory genes involved in biofilm formation in other systems. Thus, we systematically characterised genetic elements and molecular mechanisms involved in nitrogen-fixing biofilm formation. Genome-wide identification of putative genes involved in biofilm formation and mutant construction led to the identification of a complex regulatory circuitry involving the alternative sigma factors RpoN and RpoS and the Gac/Rsm regulators, and to the proposal of a model that integrates multiple levels of positive and negative regulation.

## Results

### Effect of carbon and nitrogen sources on biofilm formation and biofilm-based nitrogenase activity

**Fig. 1:**
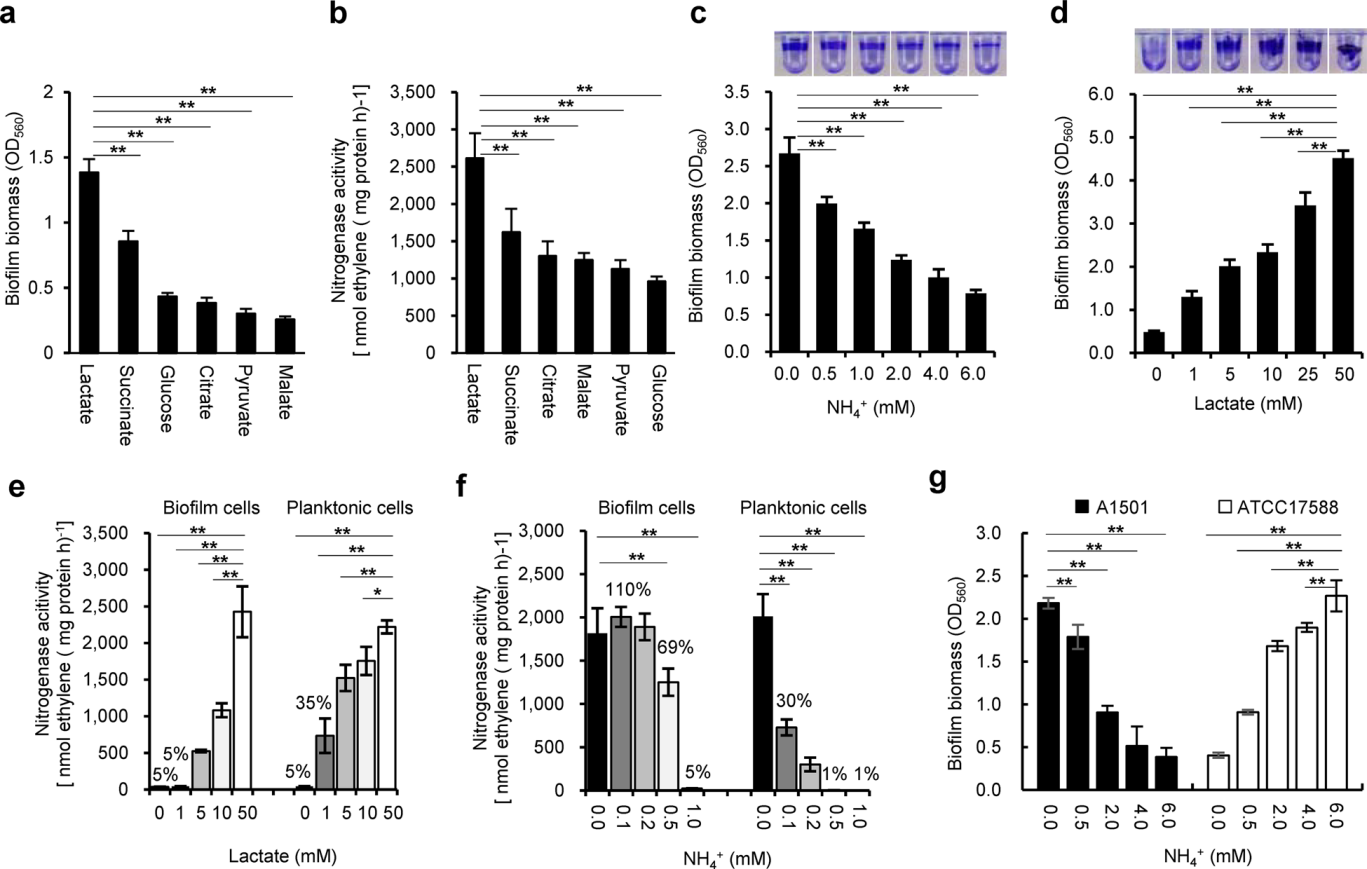
Nitrogen-fixing biofilm formation. **a** Effect of different carbon substrates on the mature biofilm biomass determined 48 h after inoculation by the crystal violet (CV) method: bacteria were inoculated in minimal medium K devoid of a nitrogen source and containing the carbon substrates at 50 mM. **b** Effect of the different carbon substrates on the nitrogenase activity under the same conditions as in **a**. **c** Effect of NH_4_^+^ concentration (0–6.0 mM) on biofilm biomass under the same conditions as in **a**. CV staining of the biofilm obtained is shown at the top. **d** Effect of lactate concentration (0–50 mM) on biofilm biomass under the same conditions as in **a**. CV staining of the biofilm obtained is shown at the top. **e** Effect of lactate concentration (0–50 mM) on nitrogenase activity under planktonic growth (with shaking at 220 r.p.m. and 0.5% oxygen) and biofilm growth conditions (without shaking in air). **f** Effect of NH_4_^+^ concentration on the nitrogenase activity of the planktonic and biofilm cells in minimal medium K containing 50 mM lactate. **g** Effect of NH_4_^+^ concentration on biofilm formation by diazotrophic and non-diazotrophic *P. stutzeri* strains in minimal medium K containing 50 mM lactate. Unlike A1501, the non-diazotrophic strain ATCC17588 favoured biofilm formation under nitrogen-sufficient conditions. Each error bar indicates the standard deviation of three independent experiments. Asterisks indicate statistical significance by one-way ANOVA with LSD multiple-comparison test: *p < 0.05; **p < 0.01.

It was previously shown that when lactate was the sole carbon source, *P. stutzeri* A1501 tended to form biofilms rather than maintain a planktonic state under nitrogen-deficient conditions^41^. To further examine this behaviour, the ability of A1501 to form mature biofilms and fix nitrogen was assayed 48 h after inoculation using carbon sources other than lactate and different concentrations of NH_4_Cl. Among the carbon substrates tested at 50 mM, lactate was the best for both biofilm formation and nitrogen fixation (Fig. 1a, b). The ability of A1501 to form biofilms gradually decreased with increasing NH_4_^+^ concentration (Fig. 1c) but was enhanced with increasing lactate concentration (Fig. 1d). In addition, ~35% of the maximum nitrogenase activity was observed in planktonic growth at a low lactate concentration (1.0 mM), but very low nitrogenase activity was detected in biofilm growth (Fig. 1e), indicating that biofilm cells were incapable of fixing nitrogen unless supplied with an adequately available carbon source. These results indicated that nitrogen-fixing biofilm growth requires a sufficient supply of carbon sources, as both biofilm formation and nitrogen fixation are energetically expensive and highly regulated processes^16,54^.

In general, the expression of nitrogenase genes can be completely inhibited by small extracellular concentrations of NH_4_Cl^49,54^. The nitrogenase activity of mature biofilms incubated without NH_4_^+^ was determined after the addition of different concentrations of NH_4_Cl (0.1 to 0.5 mM). As shown in Fig. 1f, the detectable nitrogenase activity of the planktonic cells was limited in response to the addition of 0.1 to 0.2 mM NH_4_^+^. The nitrogenase activity of biofilm cells was much higher than that of planktonic cells at low NH_4_^+^ concentrations (0.1 to 0.2 mM NH_4_^+^). In addition, 0.5 mM NH_4_^+^ caused a small reduction (31%) in nitrogenase activity of biofilm cells but almost total loss of that of planktonic cells. This result is in good agreement with the gene expression data obtained from planktonic and biofilm cells treated with different concentrations of NH_4_^+^ (Supplementary Fig. 1). A1501 biofilms were previously reported to fix nitrogen in the presence of oxygen, suggesting that the matrix could be a barrier for oxygen diffusion^41^. It also appears that the matrix could limit the inhibitory effect of NH_4_^+^ on enzyme synthesis and activity.

### Functional identification of genetic elements governing biofilm formation in A1501

**Fig. 2:**
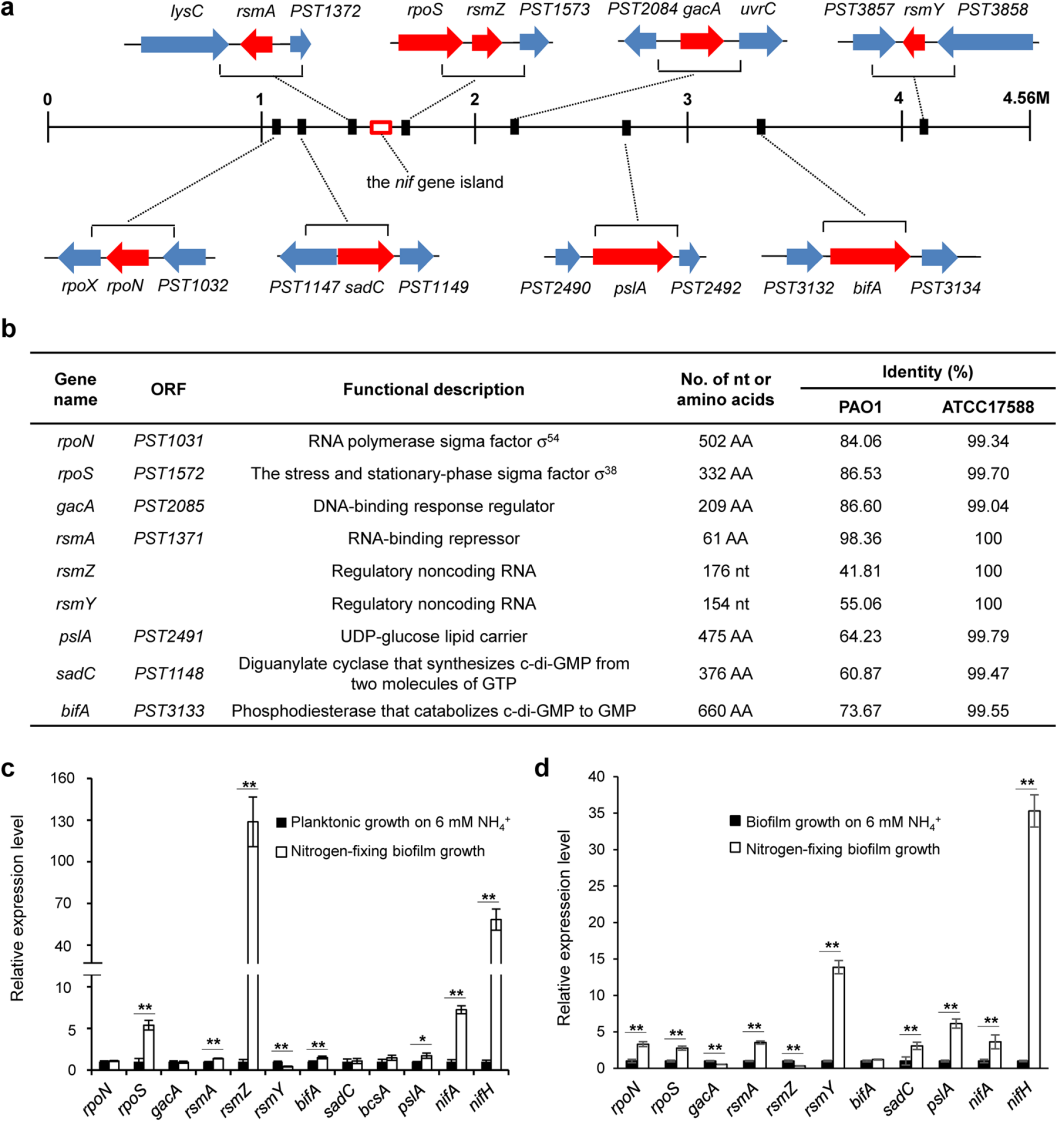
Systematic investigation of the *P. stutzeri* A1501 genes possibly involved in biofilm formation. **a** Localisation of the gene clusters on a linear map of the chromosome. The 49 kb *nif* gene island (red box) is located on the chromosome (from PST1302 to PST1359). **b** Functional description. Identities (%) shared between the amino acid sequences of orthologous proteins from *Pseudomonas aeruginosa* PAO1 and *P. stutzeri* ATCC17588 are presented. **c** Relative expression levels of the selected genes in planktonic cells on medium K containing lactate and ammonium versus nitrogen-fixing biofilm cells on medium K containing lactate. **d** Relative expression levels of the selected genes in biofilm cells on medium K containing lactate and ammonium versus nitrogen-fixing biofilm cells on medium K containing lactate. Each error bar indicates the standard deviation of three independent experiments. Asterisks indicate statistical significance by one-way ANOVA with LSD multiple-comparison test: *p < 0.05; **p < 0.01.

The A1501 genome contains a set of nine genes (namely, *gacA*, *rsmA*, *rsmY*, *rsmZ*, *sadC*, *bifA*, *pslA*, *rpoN,* and *rpoS* (Fig. 2a)), which are present in other *Pseudomonas* species and encode proteins and ncRNAs known to play roles in biofilm formation^16^ (Fig. 2b). As a working hypothesis, we suggest that 1501 may use regulatory mechanisms such as the c-di-GMP signalling and Gac/Rsm pathways common to other *Pseudomonas* species for biofilm formation. To gain insights into the potential roles of the nine genes in nitrogen-fixing biofilm formation, we monitored the gene transcription levels under nitrogen fixation conditions. In nitrogen-fixing biofilm cells, the expression of *rsmZ* showed the most dramatic increase (>120-fold), followed by that of *rpoS* (~5.0-fold) compared with their expression in planktonic state cells (Fig. 2c). In particular, the relative expression of most of these genes was superior in nitrogen-fixing biofilms than in planktonic biofilms (Fig. 2c). Indeed, the expression of genes specific for nitrogen fixation that were used as controls, such as *nifH* and *nifA*, was increased by ~35-fold and ~4.0-fold, respectively, in nitrogen-fixing biofilm cells compared to non-nitrogen-fixing biofilm cells (Fig. 2d). To establish whether these genes play a role in biofilm formation, we constructed a set of mutant strains and corresponding strains containing complementing plasmids or overexpressing the gene. We found that mutations in most of the selected genes affect biofilm formation by either decreasing or increasing biofilm formation (Table 1). These effects were not due to differences in planktonic growth, as the corresponding mutant strains displayed similar growth as wild-type (WT) A1501 in minimal medium K containing 20 mM NH_4_^+^ and 50 mM lactate (Table 1).

**Table 1:** Characteristics of the *P. stutzeri* A1501 mutant strains and the corresponding strains containing complementing plasmids or overexpressing the genes used in this study.

Strain	Biofilm formation (%)		Nitrogenase activity (%)	OD_600_^b^
	NH_4_^+^-deficient	6.0 mM NH_4_^+^		
WT A1501	100 ± 17	100 ± 20	100 ± 26	1.29 ± 0.02
A1501 (pLAFR3)^a^	96 ± 22	94 ± 10	91 ± 26	1.22 ± 0.13
Δ*gacA*	28 ± 11**	21 ± 9**	78 ± 18	1.28 ± 0.04
Δ*gacA* (pL*gacA*)	89 ± 28	84 ± 25	91 ± 30	1.22 ± 0.06
Δ*rpoN*	8 ± 0.9**	9 ± 3**	0**	1.37 ± 0.01
Δ*rpoN* (pL*rpoN*)	92 ± 9	101 ± 16	151 ± 31*	1.33 ± 0.05
Δ*rpoS*	91 ± 16	168 ± 42	75 ± 7	1.23 ± 0.03
Δ*rpoS* (pL*rpoS*)	74 ± 21	86 ± 17	89 ± 13	1.30 ± 0.06
Δ*rsmA*	152 ± 35	415 ± 72**	90 ± 22	1.33 ± 0.07
Δ*rsmA* (pL*rsmA*)	98 ± 10	173 ± 42	105 ± 18	1.13 ± 0.06
A1501 (pL*rsmA*)	76 ± 28	51 ± 7*	96 ± 29	1.08 ± 0.05
Δ*rsmZ*	35 ± 5**	50 ± 9*	63 ± 11*	1.29 ± 0.08
Δ*rsmZ* (pL*rsmZ*)	87 ± 20	90 ± 8	82 ± 16	1.24 ± 0.02
A1501 (pL*rsmZ*)	149 ± 37	167 ± 19*	92 ± 10	1.21 ± 0.04
Δ*rsmY*	69 ± 13	67 ± 6	110 ± 14	1.42 ± 0.10
Δ*rsmY* (pL*rsmY*)	96 ± 7	120 ± 19	80 ± 9	1.20 ± 0.04
A1501 (pL*rsmY*)	117 ± 15	137 ± 37	88 ± 8	1.30 ± 0.01
Δ*rsmYZ*	30 ± 5**	24 ± 11**	73 ± 16	1.24 ± 0.08
Δ*bifA*	89 ± 28	309 ± 65**	93 ± 30	1.24 ± 0.03
Δ*bifA* (pL*bifA*)	97 ± 8	119 ± 16	90 ± 4	1.36 ± 0.08
A1501 (pL*bifA*)	61 ± 6*	45 ± 15*	78 ± 22	1.20 ± 0.07
Δ*sadC*	56 ± 11*	113 ± 27	95 ± 18	1.28 ± 0.04
Δ*sadC* (pL*sadC*)	82 ± 9	106 ± 27	82 ± 7	1.25 ± 0.05
A1501 (pL*sadC*)	89 ± 6	201 ± 53*	97 ± 15	1.19 ± 0.08
Δ*pslA*	9 ± 5**	61 ± 14*	63 ± 20*	1.27 ± 0.09
Δ*pslA* (pL*pslA*)	92 ± 22	87 ± 9	118 ± 12	1.31 ± 0.10
A1501(*pLpslA*)	78 ± 3	90 ± 7	53 ± 10**	1.18 ± 0.09
Δ*nifA*	104 ± 11	97 ± 8	0**	1.28 ± 0.03
Δ*nifA* (pL*nifA*)	96 ± 12	88 ± 6	91 ± 17	1.39 ± 0.10

%: One hundred percent corresponds to the WT strain. Biofilm formation was determined 48 h after inoculation by crystal violet staining using cells grown on minimal lactate-containing medium amended with or without NH_4_Cl. Nitrogenase activity was determined using biofilm cells grown on minimal lactate-containing medium without NH_4_Cl.

^a^The empty vector control.

^b^Planktonic growth for 12 h in minimal medium K amended with 50 mM lactate and 20 mM NH_4_Cl.

All experiments were performed in three biological replicates, and the mean values (±standard deviation) are shown. Asterisks indicate statistical significance when compared to the wild-type control by one-way ANOVA with LSD multiple-comparison test: *p < 0.05; **p < 0.01.

It should be noted that mutation of the Gac/Rsm pathway genes, such as *gacA*, *rsmZ* and *RsmY*, resulted in a partial but not total loss of nitrogen-fixing biofilm production (Table 1), suggesting the involvement of additional regulatory pathways. This assumption was further confirmed by measuring the biofilm phenotypes of the strains lacking either *rpoN* or *pslA*. As shown in Supplementary Table 1, an *rpoN* mutation caused an almost total loss of biofilm production under nitrogen fixation conditions; a similar phenotype was observed in a mutant lacking the *pslA* gene, indicating that both RpoN and PslA are essential for nitrogen-fixing biofilm formation in A1501. Interestingly, the nine genes cited above (shown in Fig. 2b) are present in *P. stutzeri* ATCC17588, a non-diazotrophic strain isolated from a clinical specimen. However, when we examined the effect of nitrogen availability on biofilm formation by this strain, we found that its physiological conditions favouring biofilm formation differ from those found in A1501. Under nitrogen-sufficient conditions, the ability of this strain to form biofilms gradually increased with increasing NH_4_^+^ concentration, in contrast to the diazotrophic strain A1501, which tended to form biofilms under nitrogen-deficient conditions (Fig. 1g). A reasonable explanation for this finding is that the mechanisms underlying biofilm formation differ between diazotrophic and non-diazotrophic *P. stutzeri* strains.

### RpoN governs nitrogen-fixing biofilm formation via transcriptional activation of *pslA* and *nifA*

**Fig. 3:**
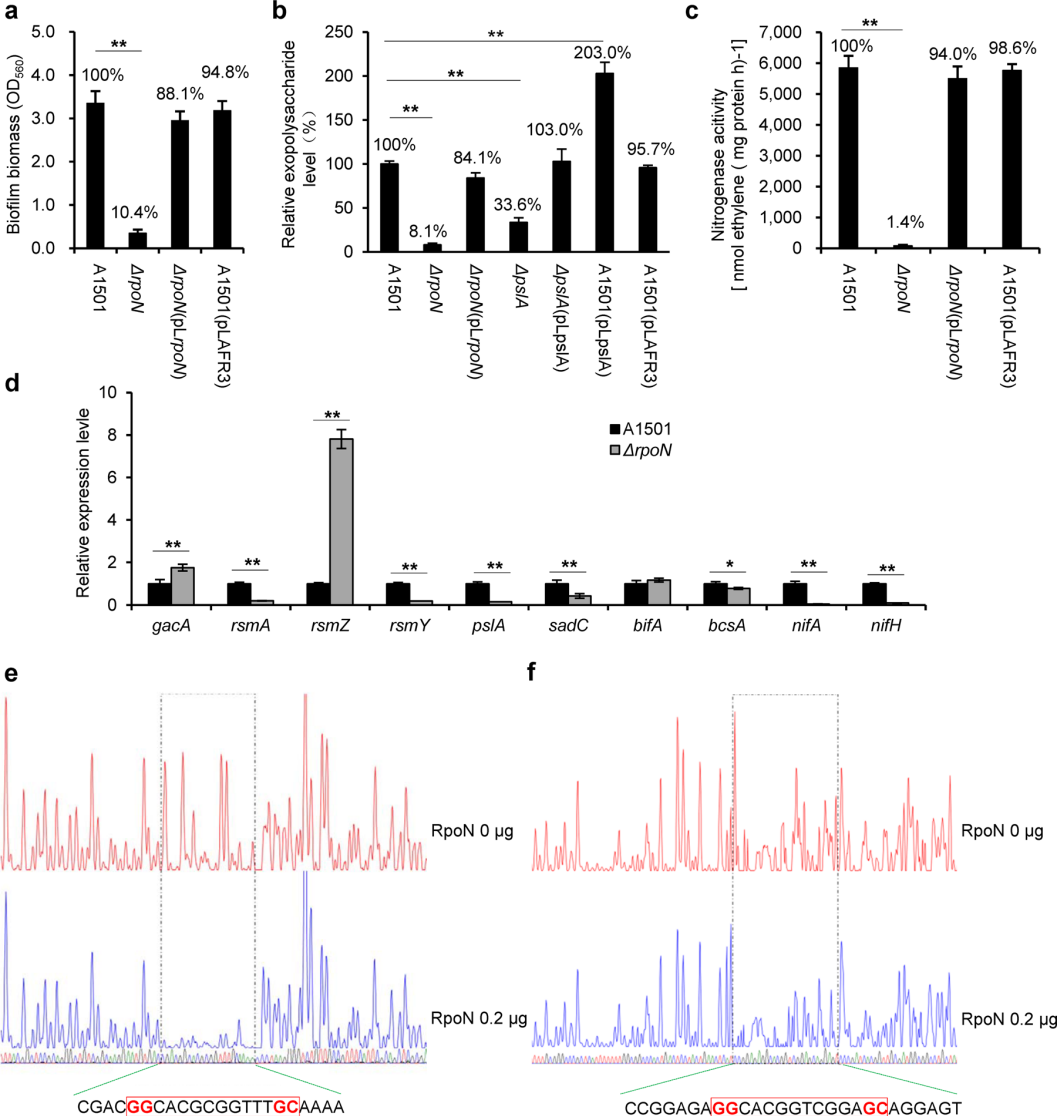
Regulation of nitrogen-fixing biofilm formation by *rpoN*. **a** Effect of *rpoN* on biofilm formation 48 h after inoculation, comparison of biofilm biomass obtained with the WT (containing or not the plasmid vector), the *rpoN* mutant and the complemented strains. **b** Relative exopolysaccharide levels in the WT (containing or not the plasmid vector), *rpoN* and *pslA* single mutant, complemented, and overexpression strains determined by HPLC analysis. **c** Effect of *rpoN* mutation on nitrogenase activity. **d** Relative expression levels of *nif* genes and biofilm-related genes in the nitrogen-fixing biofilm cells of the WT A1501 versus that of the *rpoN* mutant. Each error bar represents the standard deviation of three independent experiments. Asterisks indicate statistical significance when compared to the wild-type control by one-way ANOVA with LSD multiple-comparison test: *p < 0.05; **p < 0.01. **e**, **f** DNase I footprinting analysis of the *nifA* (**e**) and *pslA* (**f**) promot**e**r probes (300 ng) using purified RpoN protein added at 0 (upper panel) and 0.2 μg (lower panel). The RpoN-protected region is indicated by a dotted box, with the nucleotide sequence shown at the bottom. The RpoN-binding site is marked by a red box.

Mutation of *rpoN* resulted in a dramatic decrease in biofilm formation (Fig. 3a), consistent with the observation that the *rpoN* mutant was severely impaired in exopolysaccharide production (Fig. 3b). Furthermore, *rpoN* mutation led to a total loss of nitrogenase activity (Fig. 3c), suggesting that *rpoN* has a major role by controlling both the nitrogen fixation ability and the biofilm polysaccharides. Furthermore, qRT-PCR analysis provided additional evidence showing that the *rpoN* mutation affected the expression of GacA/Rsm pathway genes to different extents (Fig. 3d). In nitrogen-fixing biofilm cells, the *rpoN* mutation led to a significant decrease in the expression of *rsmA* but an increase in the expression of *gacA* and *rsmZ*, suggesting that RpoN can exert a negative effect on the expression of GacA/Rsm pathway genes. A similar phenomenon has also been reported in the *P. aeruginosa rpoN* mutant, where the expression of the *gacA* gene was significantly increased^55^. Moreover, *pslA* is the only gene among the nine genes studied (listed in Fig. 2b) to have an RpoN box-like element upstream of its transcription start site, suggesting RpoN-dependent expression, which is also the case for the *nifLA* promoter (Supplementary Fig. 2a, b). Indeed, it was determined by DNase I footprinting assays. As shown in Fig. 3e, f, RpoN protects a 22 bp DNA region (CGAC**GG**CACGCGGTTT**GC**AAAA) of the *nifLA* promoter and a 27 bp DNA region (CCGGAGA**GG**CACGGTCGGA**GC**AGGAGT) of the *pslA* promoter. Two regions overlap with the putative RpoN-binding site located at positions −12 to −24 from the transcription start. Taken together, these data suggest that the expression of both *nifA* and *pslA* genes is dependent on RpoN.

Psl is the major exopolysaccharide of the *P. aeruginosa* biofilm matrix encoded by the *psl* gene cluster, but a disruption of the first *pslA* gene of the cluster resulted in severe attenuation of Psl production^56^. The *pslA* gene found in A1501 encodes a UDP-glucose lipid carrier sharing 64% amino acid sequence identity with that of *P. aeruginosa* PAO1 (Fig. 2b). Carbohydrate monomer composition analysis of the polysaccharides from the A1501 culture showed that they were composed of glucose, mannose, galactose, ribose and rhamnose (Table 2), while those of the *pslA* mutant culture were composed mainly of glucose. Furthermore, the total amount of exopolysaccharides from the *pslA* mutant culture was 33% of the amount observed for the WT strain, whereas the exopolysaccharide-producing ability of the *pslA*-overexpressing strain was ~2.0-fold greater than that of the WT strain (Fig. 3b), indicating that PslA is a key player in the production of Psl-like exopolysaccharides. Most interestingly, the *pslA* mutant completely failed to form biofilms under nitrogen-deficient conditions but produced 64% of the biofilm that WT produced under nitrogen-rich conditions (Table 1). These results, together with the observation that *pslA* expression was significantly upregulated in nitrogen-fixing biofilm growth (Fig. 2d), favour an important role of *pslA* in Psl-like exopolysaccharide production and consequently in nitrogen-fixing biofilm formation. In addition, mutation of the *nifA* gene, encoding an activator of all *nif* genes, led to a complete loss of nitrogenase activity but had no effect on biofilm formation (Table 1). Our results indicate that RpoN-driven positive regulation at the transcriptional level is one of the key mechanisms used in diazotrophic *P. stutzeri* to govern nitrogen-fixing biofilm formation.

**Table 2:** Content and carbohydrate monomer composition of the total exopolysaccharides isolated from A1501 and the *pslA* mutant.

Strain	Exopolysaccharides		
	Content (%)	Glycosyl residue	Amount (mol %)
A1501	100	Glucose	55.1 ± 9.2
		Mannose	16.0 ± 5.4
		Galactose	11.3 ± 0.3
		Ribose	15.2 ± 5.6
		Rhamnose	2.3 ± 0.5
*pslA* mutant^a^	33.6	Glucose	95.1 ± 18.8
		Mannose	–
		Galactose	–
		Ribose	3.4 ± 1.1
		Rhamnose	1.5 ± 0.7

See ‘Methods’ section for sample preparation and analysis.

All experiments were performed in three biological replicates, and the mean values (±standard deviation) are shown.

%: One hundred per cent corresponds to the WT strain.

‘–’ Undetectable.

^a^Lost ability to produce Psl but can produce other exopolysaccharides.

### RsmA posttranscriptionally represses biofilm formation by binding *pslA* and *sadC* mRNAs

**Fig. 4:**
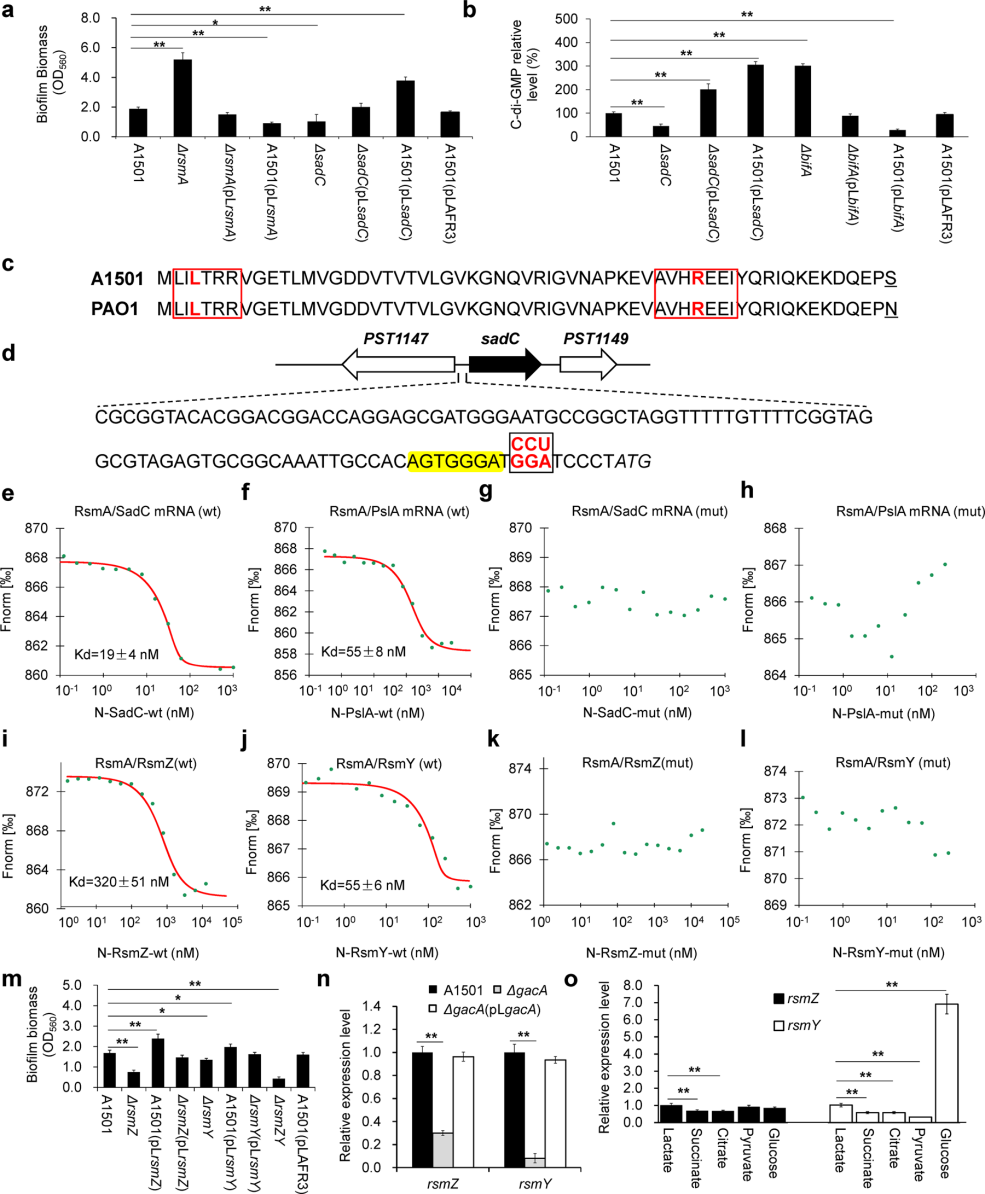
Involvement of Gac/Rsm pathway genes in biofilm formation. **a** Biofilm formation measured 48 h after inoculation by crystal violet staining of the WT A1501 and its derived strains. **b** Relative c-di-GMP levels of the WT A1501 and its derived strains. **c** Primary sequence alignment of the *P. aeruginosa* and A1501 RsmA proteins showing sequence similarities. Regions L and R, thought to mediate RNA binding, are boxed in red. **d** The *sadC* promoter region of *P. stutzeri* A1501. The GGA motif is shown in red, and the corresponding mutated sequence in the synthesised oligonucleotide N-SadC-mut, containing the 5′-UTR of *sadC* mRNA, is shown above the GGA motif. The putative ribosome-binding site is highlighted in yellow. **e**–**l** Determination of the binding affinity of Cy3-labelled RsmA to the target mRNAs or ncRNAs containing the WT or mutated GGA motifs, respectively. Point mutations introduced into synthesised oligonucleotide derivatives are shown in red. Ligand-dependent changes in MST are plotted as normalised fluorescence (*F*_norm_) values vs. ligand concentration in a dose–response curve. *F*_norm_ values are plotted as parts per thousand [‰] for binding affinity analysis. N, ssRNA oligonucleotide; wt, wild type; mut, mismatch mutation. **m**, Effects of *rsmZ* and *rsmY* deletion and overexpression on biofilm formation. **n** Effect of *gacA* mutation on the expression of *rsmZ* and *rsmY*. **o** Relative expression levels of *rsmZ* and *rsmY* in biofilm cells grown on lactate versus other carbon substrates. Asterisks indicate statistical significance by one-way ANOVA with LSD multiple-comparison test: *p < 0.05; **p < 0.01.

The presence of two RNA-binding proteins belonging to the CsrA family appears to be common in pseudomonads, e.g., RsmA and RsmE of *P. fluorescens* or RsmA and RsmF of *P. aeruginosa*^57^; however, only one gene encoding RsmA was found in the A1501 genome. In the biofilm model bacterium *P. aeruginosa*, RsmA exerts a negative effect on biofilm formation through posttranscriptional repression of the *sadC* and *pslA* genes^22,23^. As shown in Fig. 2b, the *P. stutzeri rsmA*, *pslA*, and *sadC* gene products share a high identity with orthologous proteins in *P. aeruginosa*. Analysis of mutant strains showed that deletions of each of the three genes affected the biofilm-forming ability of *P. stutzeri* A1501 to different extents (Table 1). The deletion of *rsmA* significantly enhanced biofilm formation compared with WT, whereas overexpression of this gene reduced biofilm formation (Fig. 4a), suggesting that RsmA may negatively regulate biofilm formation in A1501. Interestingly, both deletion and overexpression strains were more affected in biofilm formation under nitrogen-rich conditions than under nitrogen-deficient conditions (Table 1). As noted before, biofilm formation was strongly decreased in the *pslA* mutant under N limitation, which was correlated with a strong decrease in exopolysaccharide content (Fig. 3b). In addition, the *sadC* mutant displayed a moderate decrease in biofilm production (Fig. 4a), and its intracellular c-di-GMP level was reduced by approximately 50% compared with that of WT (Fig. 4b). In contrast, the intracellular c-di-GMP level was significantly increased in a mutant lacking *bifA*, which encodes a c-di-GMP-degrading phosphodiesterase but was decreased by *bifA* overexpression (Fig. 4b). Similar effects of *bifA* mutation and overexpression on biofilm formation were observed (Table 1).

*P. stutzeri* A1501 RsmA is predicted to be a protein of 61 amino acids, sharing ~ 99% sequence identity with the *P. aeruginosa* PAO1 RsmA (Fig. 2b). Indeed, this high similarity is reflected at the level of the L and R regions in A1501 and *P. aeruginosa* RmsA as well as other Rsm homologue proteins, including *Escherichia coli* CsrA (Csr/Rsm family), and has been reported to be involved in mRNA binding^58^. Furthermore, the 5′-UTR regions of both *pslA* (Supplementary Fig. 2a) and *sadC* (Fig. 4d) mRNAs were predicted to contain one GGA motif overlapping with the ribosome-binding site (RBS), which is generally the target of RsmA. RsmA typically represses gene expression by binding to the GGA motif and directly blocking access of the ribosome to RBS. To obtain experimental evidence for the predicted interaction, ssRNA oligonucleotides containing the GGA motifs present in the 5′-UTR of the *pslA* and *sadC* mRNAs were synthesised and incubated with increasing concentrations of fluorescently labelled RsmA. Specific interactions were quantified by microscale thermophoresis (MST). The data indicated significant binding of RsmA to the *pslA* and *sadC* mRNAs with calculated Kd values of 19 ± 4 and 55 ± 8 nM, respectively (Fig. 4e, f), whereas mutation in the GGA motifs of the *pslA* and *sadC* mRNAs completely inhibited binding with WT RsmA (Fig. 4g, h). Our results indicate that the influence on biofilm formation observed for RsmA takes place through direct repression of *pslA* and *sadC* at the posttranscriptional level.

### The RsmY and RsmZ ncRNAs competitively sequester RsmA away from its mRNA targets

Among the ncRNAs identified in the A1501 genome, two sharing identity with RsmY and RsmZ are predicted to have multiple GGA motifs located on the single-stranded outer stem loops (Supplementary Fig. 3), which is characteristic of Rsm ncRNAs able to bind the RsmA protein^19^. As expected from MST experiments, both RsmY and RsmZ bind to RsmA, exhibiting Kd values of 55 ± 6 and 320 ± 51 nM, respectively (Fig. 4i, j), and binding is abolished if the GGA motif is mutated (Fig. 4k, l). Furthermore, single deletion of either *rsmY* or *rsmZ* caused a limited reduction in biofilm production (Fig. 4m), whereas deletion of both *rsmY* and *rsmZ* resulted in a significantly decreased biofilm biomass (~76%), which was similar to the effect (~79%) observed in a *gacA* mutant (Fig. 4m and Supplementary Table 1). Consistent with the report that both Rsm ncRNAs are known to be under the control of GacA in *P. aeruginosa*^17^, the expression of both *rsmY* and *rsmZ* was strongly decreased in the A1501 *gacA* mutant (Fig. 4n). Moreover, both *rsm* genes possess a sequence corresponding to a conserved GacA-binding site in their promoters (Supplementary Fig. 3), suggesting GacA-dependent activation of the two genes.

The transcription pattern of *rsmZ* greatly differed from that of *rsmY*. For example, RsmZ expression in biofilm cells was upregulated more than 120-fold over that in the planktonic state, in contrast to RsmY, whose expression was downregulated 2.0-fold (Fig. 2c). Furthermore, the expression level of RsmZ with different carbon sources showed no significant differences, whereas the RsmY level increased ∼6.8-fold in biofilm cells grown on glucose compared to those grown on lactate, suggesting a significant induction by glucose (Fig. 4o). Overexpression of either *rsmY* or *rsmZ* led to increased biofilm production to different extents, while single mutations of *rsmY* and *rsmZ* resulted in decreased and increased levels of nitrogenase activities, respectively (Supplementary Table 1). These results suggest that RsmY and RsmZ have overlapping and different functions in A1501. Using a highly precise and absolute nucleic acid quantification technique^59^, termed droplet digital PCR (ddPCR), we further assessed the absolute copy number of the RsmA/Z/Y pool during nitrogen-fixing biofilm development. At the time of inoculation, RsmZ was present at up to ~55,000 copies per ng total RNA, much higher than RsmY and RsmA (Table 3). Most strikingly, the expression of RsmZ was upregulated rapidly (up to ~150,000) at the early stage of biofilm formation and then downregulated remarkably during biofilm maturation, suggesting that RsmZ functions as a potent trigger for the initiation of biofilm formation. In contrast, RsmY expression showed only a small upregulation. This suggests that in A1501, RsmZ but not RsmY antagonizes the posttranscriptional repression exerted by RsmA during biofilm development.

**Table 3:** Absolute transcription levels of the *rsmA, rsmY*, and *rsmZ* genes at different stages of nitrogen-fixing biofilm formation by *P. stutzeri* A1501.

Stage	Absolute transcriptional level (copies/ng total RNA)^c^		
	*rsmA*	*rsmY*	*rsmZ*
Initial inoculation	900 ± 11	978 ± 71	55,600 ± 2800
Early stage^a^	932 ± 21	1541 ± 58**	154,000 ± 9168**
Mature stage^b^	678 ± 48**	2194 ± 189**	45,733 ± 4277

^a^12 h after the initial inoculation.

^b^48 h after the initial inoculation (see ‘Methods’ section).

^c^Absolute transcription level is expressed as a number of copies per copies per ng total RNA, which was measured using droplet digital PCR.

All experiments were performed in three biological replicates, and the mean values (± standard deviation) are shown. Asterisks indicate statistical significance when compared to the initial inoculation by one-way ANOVA with LSD multiple-comparison test: *p < 0.05; **p < 0.01.

### RpoS negatively regulates biofilm formation via RsmZ under nitrogen-sufficient conditions

**Fig. 5:**
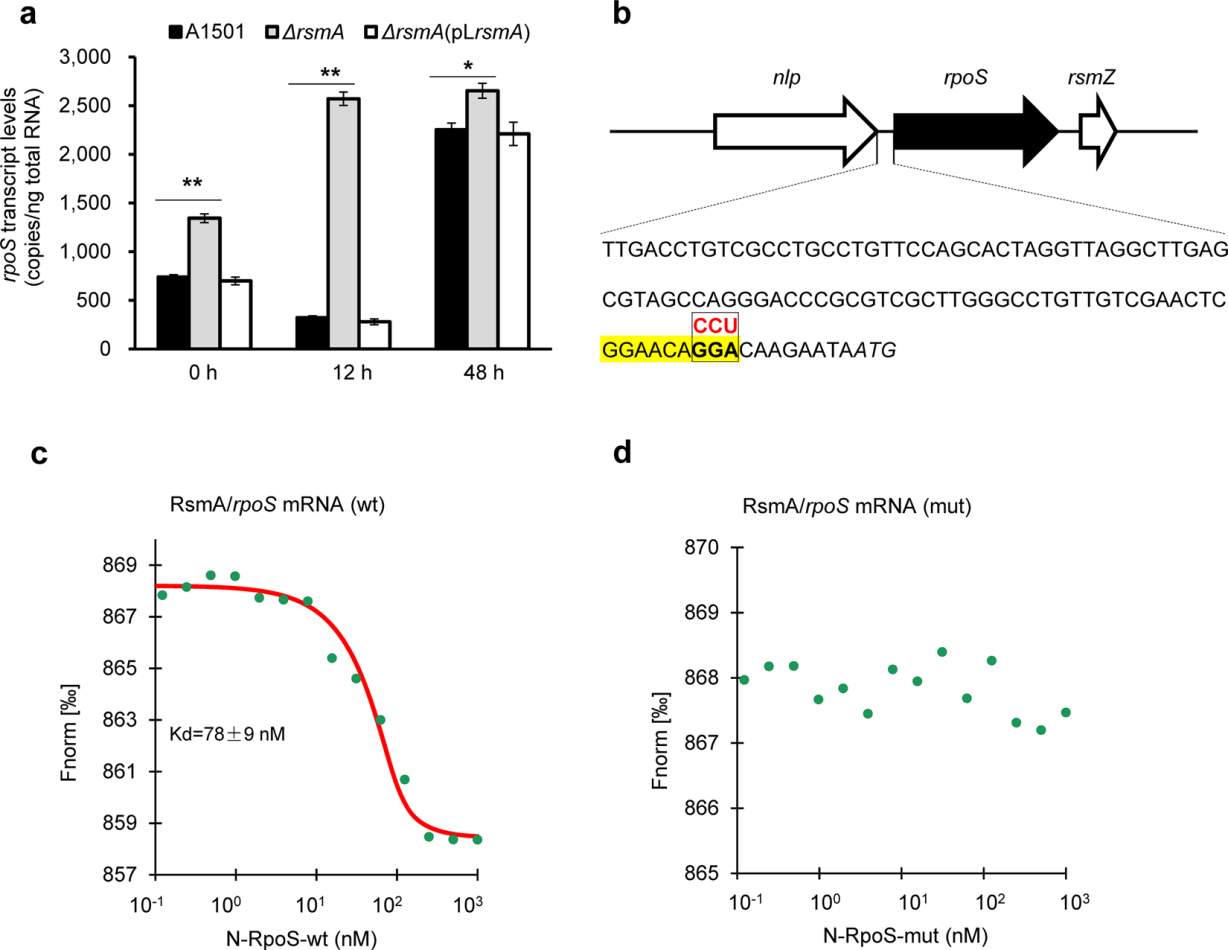
Regulation of *rpoS* expression by RsmA. **a** Absolute transcription levels of the *rpoS* gene measured using ddPCR at different stages of nitrogen-fixing biofilm formation by the WT A1501, *rsmA* mutant, and corresponding complemented strains. 0 h: initial inoculation; 12 h: early stage; 48–96 h: mature stage. Asterisks indicate statistical significance when compared to the wild-type control by one-way ANOVA with LSD multiple-comparison test: *p < 0.05; **p < 0.01. **b** The *rpoS* promoter region of *P. stutzeri* A1501. The GGA motif is shown in red, and the corresponding mutated sequence in the synthesised oligonucleotide N-RpoS-mut, containing the 5′-UTR of *rpoS* mRNA, is shown above the GGA motif. The putative ribosome-binding site (RBS) site is highlighted in yellow. The ATG initiation codon of *rpoS* is indicated in bold and italics. **c**, **d** Determination of the affinity of Cy3-labelled RsmA binding to oligonucleotides containing the WT or mutated motifs, respectively, demonstrated by MST analysis. Ligand-dependent changes in MST are plotted as normalised fluorescence (*F*_norm_) values vs. ligand concentration in a dose–response curve. *F*_norm_ values are plotted as parts per thousand [‰] for binding affinity analysis. N, ssRNA oligonucleotide; wt, wild type; mut, mismatch mutation.

The involvement of the stress and stationary-phase sigma factor RpoS in biofilm formation led to conflicting data in different bacteria^31,32^. In *P. stutzeri* A1501, the *rpoS* gene is immediately downstream of *rsmZ* (Fig. 2a), and it was shown to be involved in the regulation of two ncRNAs, *nfiS* and *nfiR*, specifically induced under nitrogen fixation conditions; therefore, the expression of both genes was downregulated in an *rpoS* mutant. In the present work, we found that the *rpoS* mutant exhibited increased biofilm production compared to that of A1501 under nitrogen-sufficient conditions (Table 1), consistent with results previously described for the *P. aeruginosa rpoS* mutant^32^ but different from the *E. coli rpoS* mutant showing decreased biofilm production^31^. Furthermore, ddPCR was used to measure the absolute transcription levels of the Gac/Rsm pathway genes during biofilm formation. As shown in Supplementary Table 4, the levels of GacA, RsmA, and RsmY showed no significant differences between the WT and *rpoS* mutant strains during biofilm development, whereas the expression of RsmZ in the *rpoS* mutant increased ~5.0-fold at the mature stage of biofilm development relative to that of the WT strain. This effect is probably indirect, as no RpoS-binding site was identified in the promoter region of the *rsmZ* gene (Supplementary Fig. 3b). These results, together with the fact that the level of *gacA* mRNA did not vary significantly in the *rpoS* mutant (Table 4), suggest that RpoS exerts an inhibitory effect on RsmZ expression in a GacA-independent manner. The ddPCR results also showed that the mRNA level of *rpoS* was very low in early-stage biofilm cells but was enhanced ~7.0-fold in mature-stage biofilm cells (Fig. 5a). In addition, the *rsmA* mutation remarkably increased the expression of *rpoS* in early-stage biofilm cells, indicating a negative effect of RsmA on *rpoS* expression (Fig. 5a). Further analysis revealed the conserved RsmA-binding GGA motif in the 5′-UTR of *rpoS* mRNA, implying a direct interaction between *rpoS* mRNA and RsmA (Fig. 5b). This possibility was checked by MST measurements, which showed that RsmA directly binds to the 5′-UTR containing the GGA motif and the ribosome-binding site of *rpoS* mRNA (Fig. 5c, d). These findings suggest a novel repression circuitry that fine-tunes biofilm development by modulating the timing and intensity of both RsmZ and RpoS expression.

**Table 4:** Absolute transcriptional levels of the *rsmA*, *rsmY*, *rsmZ*, and *gacA* genes at different stages of nitrogen-fixing biofilm formation by the *P. stutzeri* A1501 and *rpoS* mutant strains.

Stage	Absolute transcriptional level (copies/ng total RNA)^c^							
	*rsmA*		*rsmY*		*rsmZ*		*gacA*	
	A1501	*rpoS* mutant	A1501	*rpoS* mutant	A1501	*rpoS* mutant	A1501	*rpoS* mutant
Initial inoculation	740 ± 13	830 ± 8**	910 ± 23	1022 ± 89	89,003 ± 2789	84,800 ± 3959	105 ± 13	95 ± 11
Early stage^a^	798 ± 21	805 ± 23	1597 ± 154	1445 ± 110	1,388,910 ± 13,222	146,000 ± 15,900**	153 ± 17	133 ± 11
Mature stage^b^	722 ± 39	692 ± 28	634 ± 56	856 ± 92*	57,334 ± 3111	249,400 ± 28,897**	94 ± 10	75 ± 56

^a^12 h after the initial inoculation.

^b^48 h after the initial inoculation.

^c^Absolute transcriptional level is expressed as a number of copies per copies per ng total RNA, which was measured using droplet digital PCR.

All experiments were performed in three biological replicates, and the mean values (± standard deviation) are shown. Asterisks indicate statistical significance when compared to the wild-type control by one-way ANOVA with LSD multiple-comparison test: *p < 0.05; **p < 0.01.

## Discussion

**Fig. 6:**
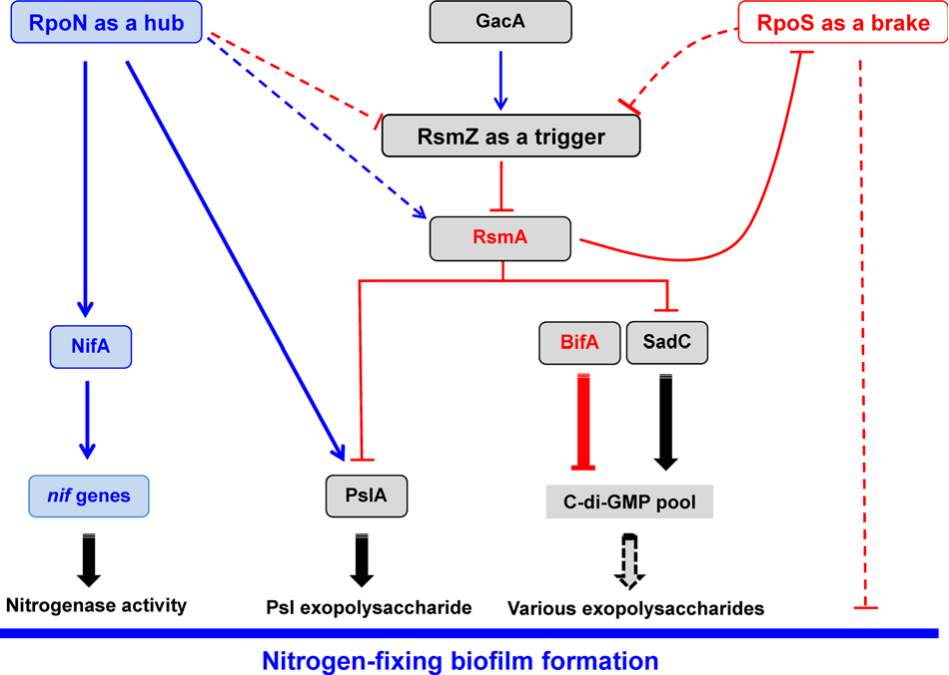
Proposed regulatory model for the *P. stutzeri* RpoN/RpoS/Gac/Rsm signal transduction systems controlling nitrogen-fixing biofilm formation at multiple levels in response to the availability of nutrients. In this model, RpoN plays a central role and may be considered a ‘hub’ to bridge nitrogen fixation and biofilm formation by activating the transcription of the *pslA* and *nifA* genes under nitrogen-deficient and carbon-sufficient conditions. RsmZ was upregulated rapidly at the early stage of biofilm formation and then downregulated remarkably during biofilm maturation, thereby acting as a potent trigger for the initiation of biofilm formation. During biofilm development, RpoS exerts a braking effect on biofilm formation by transcriptionally downregulating RsmZ expression at the mature stage; this effect is restrained by RsmA at the early stage, thereby resulting in a novel repression circuit. Additionally, RpoN likely acts as a repressor of the Gac/Rsm pathway, markedly increasing the complexity of the regulatory circuitry. Arrows and T-shaped bars indicate positive and negative regulation, respectively. Broken lines indicate direct or indirect regulations for which evidence exists but that need to be studied in further detail. The black tail arrow indicates biochemical conversion reactions. The involvement of c-di-GMP in the biosynthesis of various polysaccharides has not been demonstrated experimentally, as marked by the punctuated tail arrow. For details, refer to the text.

Numerous studies have established that regulatory circuits governing the transition from planktonic to biofilm lifestyles are very complex and differ between *Pseudomonas* species, although common regulatory mechanisms such as the c-di-GMP signalling and Gac/Rsm pathways exist. On the other hand, the available literature on the regulatory mechanisms underlying biofilm formation by nitrogen-fixing bacteria is still very scarce. Here, we aim to fill this knowledge gap by elucidating the complex mechanisms for fine-tuning nitrogen-fixing biofilm formation. In view of the data reported, we propose that multiple signalling systems regulate nitrogen-fixing biofilm formation in the rhizosphere bacterium *P. stutzeri* A1501 (as depicted in Fig. 6), including the well-studied Gac/Rsm pathway at the posttranscriptional level, RpoN-driven positive regulation at the transcriptional level, and a RpoS-mediated repression circuit at both levels. The Gac/Rsm pathway is generally considered the main mechanism controlling biofilm formation in non-diazotrophic *Pseudomonas*^15,17^. Indeed, we have shown that deletions of each of the A1501 *gac/rsm* genes can positively or negatively affect biofilm formation, but *rpoN*, by controlling the transcription of *nifA* and *plsA*, is the only gene whose inactivation resulted in the poorest biofilm and a Nif-minus phenotype. These results suggest that RpoN-driven positive regulation at the transcriptional level is one of the key mechanisms underlying nitrogen-fixing biofilm formation, which may override the effect of the Gac/Rsm pathway in diazotrophic *P. stutzeri*.

An additional level of complexity is added to this regulatory system by the presence of two structurally and functionally similar ncRNAs, RsmY and RsmZ. The presence of multiple ncRNAs with structural similarity was reported in other systems, e.g., RsmX, RsmY, and RsmZ in *P. fluorescens*^60^ and RsmW, RsmY, and RsmZ in *P. aeruginosa*^58^. These regulatory ncRNAs show similar secondary structures with numerous unpaired GGA motifs that act to sequester RsmA proteins from their targets, suggesting possible functional redundancy^20^. Since the effectiveness of ncRNA regulation is directly related to ncRNA abundance relative to their mRNA targets, this redundancy has been proposed to permit a more efficient and precise regulatory response by providing additional possibilities for integrating various signals into complex networks^18^. In the case of *P. stutzeri* A1501, a double *rsmY/rsmZ* mutation caused the same phenotypic effects on biofilm formation as those observed in the *gacA* mutant (Supplementary Table 1), suggesting that no additional Rsm ncRNAs participate in the activation of biofilm formation via the Gac/Rsm cascade in A1501. Moreover, the transcription rates of the *rsmY* and *rsmZ* genes in A1501 are clearly distinct; *rsmZ* is expressed at ~100-fold higher levels than *rsmY* under biofilm growth conditions, and the level of RsmZ is very high during biofilm growth compared to planktonic growth. In addition, a quantitative assessment by ddPCR demonstrated that RsmZ showed a biofilm stage-dependent pattern of expression with a significant increase during early stages of biofilm formation caused by transcriptional activation by GacA, which was followed by a decrease in mature biofilms. We thus propose that RsmZ rather than RsmY acts as a signal amplifier to trigger the phenotypic switch from the planktonic mode to the biofilm mode of growth. Although the biological role of RsmY is unclear at this stage, a very strong induction of *rsmY* expression by glucose was observed (Fig. 4o), implying that this ncRNA may be required for glucose-related metabolism in A1501. We also observed that single mutations of *rsmY* and *rsmZ* limit biofilm formation by A1501 but decrease and increase nitrogenase activities, respectively. These results suggest that both ncRNAs have overlapping functions in the regulation of biofilm formation but distinctive roles in the regulation of nitrogenase activity.

Our results from ddPCR experiments quantitatively show that RpoS is a mature stage-induced protein whose expression is downregulated by RsmA at the early stage of nitrogen-fixing biofilm formation. Similarly, Huertas-Rosales et al. identified *rpoS* as a target of Rsm proteins in RIP-seq experiments as an indication that RpoS regulation by Rsm proteins is direct^61^. In addition, RpoS was also found to be negatively regulated by RsmA in *P. protegens* CHA0^62^. This led us to speculate that RpoS contributes to the significant reduction in RsmZ levels when RsmZ is not needed at a high level in mature biofilm cells, while RsmA posttranscriptionally decreases RpoS expression and prevents the repression of RsmZ exerted by RpoS when RsmZ is most needed at a high level in early-stage biofilm cells. In addition to RpoS, we also found that RpoN monitors global changes in gene expression that may lead to more complex effects on nitrogen-fixing biofilm formation. For example, *rpoN* mutation significantly increased the expression of *rsmZ* in nitrogen-fixing biofilm cells, implying that RpoN likely acts as a repressor in the regulation of the RsmZ level. At least part of this effect might be mediated by GacA, as described previously in *P. aeruginosa*^55^. This appears to contradict the enhanced expression of RsmZ in nitrogen-fixing biofilm cells. However, the stronger effect of the RsmZ mutation on biofilm formation under NH_4_^+^-rich conditions than under NH_4_^+^-deficient conditions suggests that additional repression of RpoN ensures accurate and economical but not consistently high expression of RsmZ since the Gac/Rsm pathway is not the dominant player in nitrogen-fixing biofilm formation.

The initiation of biofilm formation in *P. aeruginosa* has been correlated with high intracellular levels of c-di-GMP^16^. In general, high internal levels of c-di-GMP induce the production of adhesins and extracellular matrix components, which enable bacteria to form biofilms, whereas low c-di-GMP levels lead biofilm bacteria into dispersal to shift to a planktonic mode of growth^24^. The Gac/Rsm cascade in *P. aeruginosa* is genetically linked to c-di-GMP through SadC, whose production is repressed by RsmA. We also observed a similar connection, but deletion of the *sadC* gene resulted in a strain that is partially defective in biofilm formation and c-di-GMP synthesis. This means that SadC likely contributes some but not all of the c-di-GMP under the conditions tested. Therefore, we can further infer that at least one other DGC in the A1501 genome can produce c-di-GMP. Indeed, the exact mechanism underlying c-di-GMP synthesis and biofilm formation in A1501 remains to be elucidated.

Phylogenetically close members of the *Pseudomonas* genus produce a wide diversity of exopolysaccharides, such as cellulose, Psl, and Pel^3^. The Psl polysaccharide, which is composed of mannose, glucose and rhamnose, was first described in *P. aeruginosa*^63^. Although research on Psl polysaccharides has been mostly conducted in *P. aeruginosa*, a number of *psl* gene clusters have been identified in several *Pseudomonas* strains^4^ and recently the existence of a *psl*-like gene cluster has been reported in some environmental non-aeruginosa *Pseudomonas* species^64^. Furthermore, two *P. fluorescens* strains isolated from rotted bell pepper, were previously described to produce an exopolysaccharide composed of mannose, rhamnose, and glucose substituted with pyruvate and acetate^65^. Although the exact composition of the PlsA-dependent polysaccharide, tentatively referred to as the Psl-like exopolysaccharide, is not yet established, the analysis of the glycosyl residues present in a *pslA* mutant suggests that the A1501 Psl-like exopolysaccharide contains mannose and galactose since both sugars were not found in the mutant (Table 2). From this analysis, it can be deduced that A1501 Psl differs from *P. aeruginosa* Psl, which does not contain galactose^28^. As glucose is the main sugar produced by the *pslA* mutant, it is likely that A1501 produces cellulose, in agreement with the presence of a cluster of genes in its genome that are similar to the cellulose biosynthesis genes of *P. putida* KT2440^48^. In the most recent review, Herredia-Ponce et al.^63^ stress the fact that the differences in polysaccharide composition depending on growth conditions may reflect better adaptation to specific environments due to the differential evolution that occurs in different niches.

In the case of *P. aeruginosa*, the Psl exopolysaccharide is known to be a key element at the early stage of biofilm formation and is regulated transcriptionally by RpoS^22,30^. Unlike what was observed in *P. aeruginosa*, we found that in A1501, the PlsA-dependent exopolysaccharide is essential for biofilm formation under nitrogen fixation conditions but not under nitrogen-sufficient conditions, in agreement with the fact that *pslA* transcription is RpoN-dependent. In addition to playing a major structural role in biofilms, Psl was further shown to have a signalling role in stimulating two DGCs, SiaD and SadC, to produce more of the intracellular second messenger molecule c-di-GMP^66^. A Psl-mediated increase in c-di-GMP was observed to result in two- to threefold higher levels of *pslA* transcripts, ultimately increasing the production of Psl itself and forming a unique positive feedback regulatory circuit^66^. These observations led us to speculate that PslA may be a rate-limiting enzyme of Psl synthesis. To experimentally address this possibility, pL*pslA* was introduced into A1501, generating a strain overexpressing PslA. As predicted, this overexpression strain produces much more Psl than the wild-type strain (Fig. 3c).

Bacteria in biofilms are surrounded by an extracellular matrix, which can account for up to 90% of the biofilm biomass and create a microenvironment favourable for protecting cells against various stresses^3,67^. Biofilms may provide especially suitable conditions for nitrogen fixation, as this process is extremely sensitive to oxygen and rapidly inhibited by ammonia. An early study reported that the production of exopolysaccharides under N-limiting conditions may be a survival mechanism favouring the exclusion of oxygen and increasing nitrogenase activity^68^. In addition, biofilm formation enables A1501 to fix nitrogen under aerobic conditions by forming EPS-encased cysts to protect nitrogenase from oxygen^41^. In accordance with these previous results, we found that biofilm formation was enhanced under nitrogen-deficient and carbon-sufficient conditions, which favour nitrogen fixation. Interestingly, we also observed that biofilms displayed significant nitrogenase activity at a concentration of NH_4_^+^ that completely abolished the nitrogenase activity of planktonic cells.

Nitrogen-fixing bacteria occur predominately in the rhizosphere, where carbon-rich root exudates can support the energy demands of the nitrogen fixation process, while microbial cell densities and microbial activities are the greatest, making nitrogen a key modulator of survival and competitiveness. The colonisation of the root rhizosphere is an essential step in the establishment of efficient nitrogen-fixing associations, and thus, understanding the mechanism of biofilm formation is of major interest. In the present work, we found that conditions favouring biofilm formation differ between the diazotrophic and non-diazotrophic *P. stutzeri* strains, although both strains contain the same set of Gac/Rsm and c-di-GMP signalling pathway genes, reflecting the differential evolution of their regulatory networks due to different physiologies and niches. We hypothesised that variations in biofilm phenotypes could be due to differences in transcriptional regulation. However, we found no significant differences in the putative promoter sequences of the genes listed in Fig. 2b between the two strains, suggesting that the mechanism that causes the biofilm phenotypes of the two strains to differ is much more complex than we initially believed. In addition, it is not surprising that with evolutionary optimisation in the rice rhizosphere, A1501 has evolved sophisticated regulatory networks to respond to multiple environmental cues and adapt to the environmental conditions of the rhizosphere. Of particular note is that RpoN, an alternative sigma factor typically associated with general nitrogen responses in bacteria, was found to act as a critical regulatory hub to activate the transcription of *pslA* and *nifA*, consequently forming a novel regulatory link between nitrogen fixation and biofilm formation. This regulation is probably more direct and efficient than the Gac/Rsm regulatory cascades widely found in *Pseudomonas*, and is likely advantageous, especially when diazotrophs face competition from other species in nitrogen-limited environments, such as the rhizosphere. To our knowledge, this is the unique example of multiple regulatory networks governing the transition from the planktonic mode to the nitrogen-fixing biofilm mode, which may contribute to diazotrophic *P. stutzeri* being highly adaptable to nitrogen-poor environments and have implications for the control of biofilm-related interactions between diazotrophs and host plants. Our results provide a basis for understanding a regulatory mechanism including RpoN, RpoS, Gac and Rsm regulators that underlies nitrogen-fixing biofilm development and may be applicable to various diazotrophic species. As nitrogen-fixing bacteria are found ubiquitously in most ecosystems and widely used as biofertilizers worldwide, our systematic study of nitrogen-fixing biofilms will be of both ecological and biotechnological importance.

## Methods

### Bacterial strains, plasmids and growth conditions

The bacterial strains and plasmids used in this study are listed in Supplementary Table 1. *P. stutzeri* A1501 and its derivatives were grown on LB medium or minimal medium K (containing 0.4 g l^−1^ KH_2_PO_4_, 0.1 g l^−1^ K_2_HPO_4_, 0.1 g l^−1^ NaCl, 0.2 g l^−1^ MgSO_4_·7H_2_O, 0.01 g l^−1^ MnSO_4_·H_2_O, 0.01 g l^−1^ Fe_2_(SO_4_)_3_·H_2_O, and 0.01 g l^−1^ Na_2_MoO_4_·H_2_O, pH 6.8) supplemented by the desired carbon and nitrogen sources at concentrations indicated in the text. Unless stated otherwise, growth experiments were conducted using medium K containing NH_4_Cl (20 mM) and sodium lactate (50 mM) as the sole nitrogen and carbon sources at 30 °C under vigorous shaking in a water-bath shaker. For measurements of biofilm formation, nitrogenase activity and gene expression, the concentrations of carbon substrates were adjusted to 50 mM. Antibiotics were used at the following concentrations: 50 μg ml^−1^ ampicillin (Amp), 50 μg ml^−1^ kanamycin (Km), 10 μg ml^−1^ tetracycline (Tc), 34 μg ml^−1^ chloromycetin (Cm), and 20 μg ml^−1^ gentamicin sulfate (Gm).

### Constructions of mutants, complementing plasmids and overexpression strains

The plasmids and oligonucleotide primers used in this study are listed in Supplementary Tables 1 and 2, respectively. Strains and plasmids were constructed using conventional techniques. Nonpolar insertion mutant strains (e.g., *rsmA*) were generated by homologous suicide plasmid integration as described previously^50^. Appropriate oligonucleotide primers were designed to generate amplicons that were cloned into pK18mob as a vector^69^, and the resulting plasmids were introduced into A1501 by triparental mating using pRK2013^70^, generating the mutant strains. Correct recombination was confirmed by PCR followed by nucleotide sequencing of the amplicons obtained.

To generate nonpolar deletion mutant strains, amplification of DNA fragments located upstream and downstream of the target gene was performed using the appropriate primer sets upF/upR and downF/downR (Supplementary Table 2). Then, both amplicons and a DNA fragment containing the chosen resistance cassette gene were fused, and the resulting fragment was cloned into the pK18mob*sacB* vector, as depicted in Supplementary Fig. 4. The resulting plasmid was then introduced into A1501 by triparental mating as described above, and double recombination was selected on the basis of sucrose resistance. Correct recombination was validated by PCR and sequencing using primers testF and testR.

The complemented and overexpression strains were constructed using the broad host plasmid pLAFR3. A DNA fragment containing a WT gene (e.g., *gacA*) with its promoter and terminator was amplified from genomic DNA of A1501 and cloned into pLAFR3. The resulting complementing plasmid was then introduced into the WT or mutant strain by triparental mating, generating overexpression and complemented strains, respectively (Supplementary Table 1). The gene expression levels of the overexpression strains were confirmed to be higher than those of the WT using qRT-PCR.

The mutant with both *rsmZ* and *rsmY* deleted was constructed using *ΔrsmZ* as the starting strain. Briefly, a 1154 bp fragment containing the Gm resistance cassette located between the upstream and downstream DNA fragments of *rsmY* was generated by overlap extension PCR, double-digested with *Bam*HI/*Hin*dIII, and then cloned into the *Bam*HI/*Hin*dIII site of pK18mob*sacB*. The resulting plasmid, pK18*rsmY*, was introduced into the genome of *ΔrsmZ* by triparental mating and double recombination was selected on the basis of sucrose resistance. Correct recombination in the resulting *ΔrsmZ ΔrsmY* double mutant was checked by PCR using the primers M-*rsmY*(up)-F and M-*rsmY*(down)-R (Supplementary Table 2), followed by nucleotide sequencing of the obtained PCR products. The resulting double deletion mutant (Supplementary Table 1) was used for further study.

### Biofilm formation assays

Surface-adhered biofilm formation was assayed using the crystal violet (CV) method and performed in 96-well microtiter plates. Strains used for biofilm experiments were grown overnight in LB at 30 °C. Cultures were centrifuged and diluted to a final OD_600_ of 0.2 in fresh minimal medium K containing different carbon sources with or without 6 mM NH_4_Cl. Two hundred μl of each culture was aliquoted into separate wells in a 96-well PVC plate. Microtiter plates were carefully wrapped using parafilm and placed in a 30 °C incubator without agitation for 12 or 48 h. In this study, the so-called early- and mature-stage biofilms were defined as biofilms formed 12 and 48 h after inoculation, respectively. At the indicated points, nonadhered planktonic cells were removed using a multichannel pipette without disturbing the biofilm area, and individual wells were washed twice with 160 μl of sterile double-distilled H_2_O. Then, 160 μl of 0.1% CV solution in ethanol was added to each well for 10 min and washed four times with 200 μl of ddH_2_O. Photos were taken, and the cell-associated CV was solubilized with 30% acetic acid and quantified by measuring the OD_560_ of the resulting solution using a spectrophotometer (Thermo Scientific).

### Nitrogenase activity assays

Nitrogenase activity was determined according to a previous protocol with modifications^71^. To examine the nitrogenase activity of cells grown planktonically, cells from an overnight culture in LB medium were centrifuged and resuspended in a 60 ml flask containing 10 ml of minimal N-free and lactate-containing medium at an OD_600_ of 0.1. The suspension was incubated for 4 h at 30 °C with vigorous shaking under an argon atmosphere containing 0.5% oxygen, and then 10% acetylene was added. Gas samples (0.25 ml) were taken at regular intervals (4, 6, 8, and 10 h) to determine the amount of ethylene produced. Samples were analysed on a polydivinylbenzene porous bead GDX-502 column using an SP-2100 gas chromatograph fitted with a flame ionisation detector (Beijing Beifen-Ruili Analytical Instrument Co., Ltd.). The ethylene content in the gas samples was determined by reference to an ethylene standard.

To determine the biofilm-based nitrogenase activity, strains used were grown overnight in LB at 30 °C. Cultures were centrifuged and diluted to a final OD_600_ of 0.2 in a 60 ml flask containing 10 ml of minimal NH_4_^+^-free and lactate-containing medium. The suspension was incubated for 48 h at 30 °C under static conditions in air, and then 10% acetylene was added. Gas samples (0.25 ml) were taken at regular intervals (4, 6, 8, and 10 h) to determine the amount of ethylene produced. A 4 h incubation time was chosen for qRT-PCR or ddPCR assays of biofilm-related gene expression.

To examine the effect of NH_4_^+^ on nitrogenase activity, both 10% acetylene and ammonium at different concentrations were added to 60 ml flasks containing suspensions of either biofilm or planktonic cells, and then gas samples (0.25 ml) were taken at regular intervals (4, 6, 8, and 10 h) to determine the amount of ethylene produced using the same method as described above. The nitrogenase activity was expressed as nmol ethylene min^−1^ mg^−1^ protein. Protein concentrations were determined using the Bio-Rad protein assay reagent kit (Bradford, Bio-Rad).

### RNA isolation and qRT-PCR assays

Total RNA was isolated with an innuPREP RNA Mini Kit (Analytik Jena) according to the manufacturer’s instructions. For quantification of gene expression, total RNA was reverse transcribed using random primers and the High Capacity cDNA Transcription Kit (Applied Biosystems) according to the manufacturer’s instructions. PCR was carried out with Power SYBR Green PCR Master Mix on an ABI Prism 7500 Sequence Detection System (Applied Biosystems) according to the manufacturer’s recommendations. The 16S rRNA gene was used as the endogenous reference control, and relative gene expression was determined using the comparative threshold cycle 2^−ΔΔCT^ method. Data were analysed using ABI PRISM 7500 Sequence Detection System Software (Applied Biosystems). Primers were designed based on the full genome sequence of *P. stutzeri* A1501, and they are listed in Supplementary Table 2.

### Absolute quantification of RNA copy number by droplet digital PCR (ddPCR)

Total RNA isolation and reverse transcription were performed as described above for qRT-PCR. Quantification by ddPCR was carried out in 20 μl reactions containing 10 μl of QX200 ddPCR EvaGreen SuperMix, 250 nM each commercial probe, 900 nM specific commercial primers, and 1 μl of cDNA according to the manufacturer’s recommendations. A negative control contained sterile double-distilled water only. Emulsified 1 nl reaction droplets were generated using a QX100 droplet generator (Bio-Rad) and a droplet generator DG8 cartridge (Bio-Rad) containing 20 μl of reaction mixture and 70 μl of ddPCR droplet generation oil (Bio-Rad) per well. Thirty-five μl of the generated droplet emulsions was transferred to 96-well PCR plates that were then heat-sealed using foil sheets. Target DNA amplification was performed by thermal cycling of the droplet emulsions as follows: initial denaturation at 95 °C for 10 min; 40 cycles of 94 °C for 30 s and 60 °C for 1 min; and then 98 °C for 10 min. The fluorescence of each thermal cycled droplet was measured using a QX100 droplet reader (Bio-Rad). Data were analysed using QuantaSoft software (Bio-Rad) after setting a threshold using the fluorescence of negative controls.

### 5′ Rapid amplification of cDNA ends to determine transcriptional start sites

The transcriptional start site of the six target genes (*pslA*, *nifLA*, *rpoS*, *sadC*, *rsmZ*, and *rsmY*) was determined using the rapid amplification of cDNA ends (5′ RACE) method (Invitrogen) following the manufacturer’s instructions. Briefly, the first-strand cDNA was synthesised using the primer GSP1, which was specific for the target gene sequence. The purified cDNA was tailed with dCTP by terminal deoxynucleotidyl transferase. PCR amplification was performed using the sequence-specific primer GSP2 and the anchor primer AAP. Primers GSP1 and GSP2, specific for the target gene tested here, are listed in Supplementary Table 2. The 5′ RACE products were cloned into the pGEM-T Easy vector (Promega) and sequenced to map the 5′ end of the transcript.

### Expression and purification of RsmA for microscale thermophoresis (MST) measurements

The RsmA protein was expressed and purified using the IMPACT^TM^ (Intein Mediated Purification with an Affinity Chitin-binding Tag) system according to the manufacturer’s instructions (New England Biolabs). To this end, a fragment of *rsmA* was amplified by PCR using the pTWIN1-rsmA-F/R primers (Supplementary Table 2). The PCR product was digested with *Nde*I and *Eco*RI and ligated into the protein expression vector pTWIN1, which had been digested with the same enzymes. The resulting plasmid (named pTWIN1-RsmA) was introduced into the *E. coli* BL21 (DE3) strain. Overproduction of the RsmA-intein fusion was induced by the addition of 0.5 mM isopropyl β-d-1-thiogalactopyranoside (IPTG) to cells grown to mid-log phase (OD_600_ of 0.6). Cleavage of the RsmA-intein fusion was induced by equilibrating the chitin beads in buffer B3 (1 mM EDTA, 40 mM DTT, 20 mM Tris-HCl, 500 mM NaCl, pH 8.5) overnight. The untagged RsmA was eluted from the column and dialysed against 20 mM Tris-HCl and 500 mM NaCl, pH 8.0. The purity of the protein was as high as 90%, as judged by SDS-Tris-glycine PAGE. RsmA was quantitated using the Bio-Rad protein assay reagent kit (Quick Start Bradford, Bio-Rad) and stored at −80 °C for further use.

### MST measurements to determine interactions between the RsmA protein and its target RNAs

MST experiments were performed as previously described^72^. DNA templates carrying *rsmY*, *rsmZ*, *sadC*, *pslA*, and *rpoS* bearing point mutations within the GGA sequences were amplified using the WT or mutagenic primers (Supplementary Table 2). The following transcripts were synthesised from PCR-generated templates by GenePharma using a MAXIscript kit (Thermo Fisher): the full-length WT or mutated ncRNAs (RsmY and RsmZ) and the WT or mutated oligonucleotides containing the 5′-UTR and the first 50 nucleotides of the coding sequences of *sadC*, *pslA*, and *rpoS* mRNAs. The RsmA protein was labelled with NT-647-NHS dye using the Monolith Labelling Kit RED-NHS (no. MO-L011, NanoTemper Technologies) according to the manufacturer’s instructions. For the measurements, the concentration of the labelled RsmA protein was kept constant (20 nM), while the concentrations of non-labelled ssRNA oligonucleotides varied from 0.3 nM to 10 μM. The binding reactions were carried out in MST buffer (10 mM HEPES pH 7.4, 150 mM NaCl, 10 mM MgCl_2_) supplemented with 0.1% Tween. The reactants were initially incubated at 37 °C for 30 min to enable ssRNA binding with RsmA. The samples were then loaded onto NT.115 standard capillaries (no. MO-K002, NanoTemper Technologies). The measurements were carried out at 25 °C with 40% excitation power and medium MST power. Data analyses were performed with NanoTemper Analysis software (NanoTemper Technologies).

### Purification of His-tagged RpoN for DNase I footprinting assays

The pET-28a expression system (Novagen) was used to produce C-terminally His-tagged RpoN within host *E. coli* BL21 (DE3) cells. A fragment of *rpoN* was amplified by PCR using the pET28a-*rpoN*-F/R primers (Supplementary Table 2). The PCR product was digested with *Nde*I and *Hin*dIII and ligated into the protein expression vector pET28a, which had been digested with the same enzymes. The resulting plasmid (pET28a-RpoN) was introduced into the *E. coli* BL21 (DE3) strain. An overnight culture of BL21 (DE3) harbouring the expression plasmid was used to inoculate LB medium containing the appropriate antibiotics. This cell culture was incubated with shaking at 37 °C until the OD_600_ was 0.6–0.9, at which point production of His-tagged RpoN was induced by the addition of IPTG to a final concentration of 0.1 mM. His-tagged RpoN was purified using a Ni-NTA Fast Start Kit (Qiagen, Venlo, Netherlands) according to the manufacturer’s instructions. The purity of the protein was as high as 90%, as judged by SDS-Tris-glycine PAGE. His-tagged RpoN was quantitated using the Bio-Rad protein assay reagent kit (Quick Start™ Bradford, Bio-Rad) and stored at −80 °C for further use.

### DNase I footprinting assays

DNase I footprinting assays were performed by Tolo Biotech according to a method previously described^73^. The DNA probe was prepared by PCR-amplifying a 296-bp *nifA* promoter region using the primers FP-*nifA*-F/FP-*nifA*-R and a 279-bp *pslA* promoter region using the primers FP-*pslA*-F/FP-*pslA*-R (Supplementary Table 2). For each assay, 300 ng probes were incubated with 0.2 μg purified protein RpoN in a total volume of 40 µl. After incubation for 30 min at 30 °C, 10 µl solution containing ~0.015 units DNase I (Promega) and 100 nmol freshly prepared CaCl_2_ was added and further incubation was performed at 37 °C for 1 min. The reaction was stopped by adding 140 µl DNase I stop solution (200 mM unbuffered sodium acetate, 30 mM EDTA and 0.15% SDS). Samples were first extracted with phenol/chloroform, and then precipitated with ethanol. Pellets were dissolved in 30 µl MiniQ water. The preparation of the DNA ladder, electrophoresis and data analysis were the same as previously described^73^, except that the GeneScan-LIZ600 size standard (Applied Biosystems) was used.

### Exopolysaccharide isolation and carbohydrate monomer composition analysis

Bacterial aerated cultures were grown in minimal medium K containing 50 mM lactate and 6.0 mM NH_4_Cl. Then, the cultures were centrifuged. The supernatants were collected and concentrated to 50 ml with a CentriVap concentrator (Labconco, USA). Exopolysaccharides were isolated from culture supernatants by the addition of two volumes of chilled absolute ethanol, and then proteins were removed from the exopolysaccharides by protease hydrolysis. After further precipitation by cold absolute ethanol and lyophilization, purified exopolysaccharides were obtained. The exopolysaccharide content of samples was monitored quantitatively by using the phenol-sulfuric acid method and was further normalised to the total cell protein remaining after extraction, as determined using the Bio-Rad protein assay reagent kit (Quick Start™ Bradford, Bio-Rad). Final concentrations were expressed as mg exopolysaccharide per g bacterial protein.

Carbohydrate monomer composition analysis was conducted on the exopolysaccharide samples from the A1501 and *∆pslA* mutant strains at Beijing Ketian Technology Co., Ltd. Briefly, an exopolysaccharide sample (2.0 mg) was dissolved in 5 ml of trifluoroacetic acid (2 M) and subsequently hydrolysed at 120 °C for 4 h. After repeated rotary evaporations to completely remove the trifluoroacetic acid, the sample was dissolved in 2 ml of deionized water. The hydrolysate (100 µl) was placed in a separate tube with 10 µl of deuterium-labelled succinic acid (1.5 mg ml^−1^) as the internal standard and then lyophilised. Methyl glycosides were prepared from the dry sample by suspension in 50 µl of methoxyammonium hydrochloride/pyridine solution (20 mg ml^−1^) at 40 °C for 80 min. The resulting sample was then per-*O*-trimethylsilylated with *N*-methyl-*N*-(trimethylsilyl) trifluoroacetamide (80 µl) in a water-bath pot at 40 °C for 80 min. The sample was centrifuged at 12,000 r.p.m. for 5 min. The supernatant fraction was filtered through a 0.22 µm vacuum filter and collected for glycosyl composition analysis. Samples were analysed using a gas chromatograph coupled to a mass selective detector (7890 A/5975 C MSD; Agilent Technologies, Inc.) equipped with an HP-5 (30 m by 0.32 mm, 0.25-µm film thickness; SGE Analytical Science) capillary column. The injector and detector temperatures were 250 °C and 240 °C, respectively. The column pressure was kept at 0.10 MPa, with a 1.0 ml/min carrier gas (N_2_) flow rate. The chemical compounds were identified using a mass spectral library (NIST 08) and Agilent GC-MS Workstation software. Data from three biological replicates were analysed for each strain.

### Determination of the intracellular c-di-GMP concentrations

Intracellular c-di-GMP was extracted from the indicated strains, and then the samples were analysed using reversed-phase-coupled HPLC-tandem mass spectrometry (MS/MS) as previously described^74^. Briefly, the number of cells for initial extraction was adjusted to the equivalent of 10 ml of culture with an OD_600_ of 1.0. Following three cycles of heat and ethanol extractions, the supernatants were combined, dried using a Speed-Vac, and resuspended in 200 μl of water. Samples were analysed by reversed-phase-coupled HPLC-MS/MS (Agilent). Chromatographic separation was performed on an Agilent 1260 Series HPLC system with a reverse-phase C18 column (Eclipse plus C18 RRHD, 1.8 µm). Samples (2 µl) were injected and analysed at a flow rate of 0.2 ml/min with the following gradient: 0–5 min 95% A to 70% A, 5–7 min 70% A to 5% A, and 7–24 min 95% A [buffer A: 10 mM ammonium acetate and 0.1% (v/v) acetic acid; buffer B, methanol]. Analyte detection was performed on an Agilent G6400 series triple quadrupole mass spectrometer. Commercially available c-di-GMP (InvivoGen) was used as a reference for the identification and quantification of c-di-GMP in cell extracts. Intracellular c-di-GMP content was further normalised to the total cell protein remaining after extraction, as determined using the Bio-Rad protein assay reagent kit (Quick Start™ Bradford, Bio-Rad). Final concentrations were expressed as pmol c-di-GMP per mg bacterial protein. Data from three biological replicates were analysed for each strain.

### Reporting summary

Further information on research design is available in the Nature Research Reporting Summary linked to this article.

## Supplementary information

Supplementary Information

Reporting Summary

## Data Availability

The data that support the findings of this study are available in the Supplementary Material of this article.

## References

[1] Stoodley P, Sauer K, Davies DG, Costerton JW (2002). Biofilms as complex differentiated communities. Annu. Rev. Microbiol..

[2] Morris CE, Monier JM (2003). The ecological significance of biofilm formation by plant-associated bacteria. Annu. Rev. Phytopathol..

[3] Flemming HC, Wingender J (2010). The biofilm matrix. Nat. Rev. Microbiol..

[4] Mann EE, Wozniak DJ (2012). *Pseudomonas* biofilm matrix composition and niche biology. FEMS Microbiol. Rev..

[5] Oliveira NM (2015). Biofilm formation as a response to ecological competition. PLoS Biol..

[6] Flemming HC (2016). Biofilms: an emergent form of bacterial life. Nat. Rev. Microbiol..

[7] Hall-Stoodley L, Costerton JW, Stoodley P (2004). Bacterial biofilms: from the natural environment to infectious diseases. Nat. Rev. Microbiol..

[8] Kassinger SJ, van Hoek ML (2020). Biofilm architecture: an emerging synthetic biology target. Synth. Syst. Biotechnol..

[9] Brandwein M, Steinberg D, Meshner S (2016). Microbial biofilms and the human skin microbiome. npj Biofilms Microbiomes.

[10] Lee K, Yoon SS (2017). *Pseudomonas aeruginosa* biofilm, a programmed bacterial life for fitness. J. Microbiol. Biotechnol..

[11] Shirtliff ME, Mader JT, Camper AK (2002). Molecular interactions in biofilms. Chem. Biol..

[12] Southey-Pillig CJ, Davies DG, Sauer K (2005). Characterization of temporal protein production in *Pseudomonas aeruginosa* biofilms. J. Bacteriol..

[13] Heacock-Kang Y (2017). Spatial transcriptomes within the *Pseudomonas aeruginosa* biofilm architecture. Mol. Microbiol..

[14] Varadarajan AR (2020). An integrated model system to gain mechanistic insights into biofilm-associated antimicrobial resistance in *Pseudomonas aeruginosa* MPAO1. npj Biofilms Microbiomes.

[15] Mikkelsen H, Sivaneson M, Filloux A (2011). Key two-component regulatory systems that control biofilm formation in *Pseudomonas aeruginosa*. Environ. Microbiol..

[16] Fazli M (2014). Regulation of biofilm formation in *Pseudomonas* and *Burkholderia* species. Environ. Microbiol..

[17] Brencic A, Lory S (2009). Determination of the regulon and identification of novel mRNA targets of *Pseudomonas aeruginosa* RsmA. Mol. Microbiol..

[18] Chambers JR, Sauer K (2013). Small RNAs and their role in biofilm formation. Trends Microbiol..

[19] Lapouge K (2013). RNA pentaloop structures as effective targets of regulators belonging to the RsmA/CsrA protein family. RNA Biol..

[20] Janssen KH (2018). Functional analyses of the RsmY and RsmZ Small Noncoding regulatory RNAs in *Pseudomonas aeruginosa*. J. Bacteriol..

[21] Dubey AK, Baker CS, Romeo T, Babitzke P (2005). RNA sequence and secondary structure participate in high-affinity CsrA-RNA interaction. RNA.

[22] Irie Y (2010). *Pseudomonas aeruginosa* biofilm matrix polysaccharide Psl is regulated transcriptionally by RpoS and post-transcriptionally by RsmA. Mol. Microbiol..

[23] Moscoso JA (2014). The diguanylate cyclase SadC is a central player in Gac/Rsm-mediated biofilm formation in *Pseudomonas aeruginosa*. J. Bacteriol..

[24] Valentini M, Filloux A (2016). Biofilms and cyclic di-GMP (c-di-GMP) signaling: lessons from *Pseudomonas aeruginosa* and other bacteria. J. Biol. Chem..

[25] Merritt JH, Brothers KM, Kuchma SL, O’Toole GA (2007). SadC reciprocally influences biofilm formation and swarming motility via modulation of exopolysaccharide production and flagellar function. J. Bacteriol..

[26] Rodesney CA (2017). Mechanosensing of shear by *Pseudomonas aeruginosa* leads to increased levels of the cyclic-di-GMP signal initiating biofilm development. Proc. Natl Acad. Sci. USA.

[27] Borlee BR (2010). *Pseudomonas aeruginosa* uses a cyclic-di-GMP-regulated adhesin to reinforce the biofilm extracellular matrix. Mol. Microbiol..

[28] Franklin MJ, Nivens DE, Weadge JT, Howell PL (2011). Biosynthesis of the *Pseudomonas aeruginosa* extracellular polysaccharides, alginate, Pel, and Psl. Front. Microbiol..

[29] Jackson KD, Starkey M, Kremer S, Parsek MR, Wozniak DJ (2004). Identification of *psl*, a locus encoding a potential exopolysaccharide that is essential for *Pseudomonas aeruginosa* PAO1 biofilm formation. J. Bacteriol..

[30] Overhage J, Schemionek M, Webb JS, Rehm BHA (2005). Expression of the *psl* operon in *Pseudomonas aeruginosa* PAO1 biofilms: PslA performs an essential function in biofilm formation. Appl. Environ. Microbiol..

[31] Adams JL, McLean RJC (1999). Impact of *rpoS* deletion on *Escherichia coli* biofilms. Appl. Environ. Microbiol.

[32] Xu KD, Franklin MJ, Park CH, McFeters GA, Stewart PS (2001). Gene expression and protein levels of the stationary phase sigma factor, RpoS, in continuously-fed *Pseudomonas aeruginosa* biofilms. FEMS Microbiol. Lett..

[33] Corona-Izquierdo FP, Membrillo-Hernandez J (2002). A mutation in *rpoS* enhances biofilm formation in *Escherichia coli* during exponential phase of growth. FEMS Microbiol. Lett..

[34] Schuster M, Hawkins AC, Harwood CS, Greenberg EP (2004). The *Pseudomonas aeruginosa* RpoS regulon and its relationship to quorum sensing. Mol. Microbiol..

[35] Sapi E, Theophilus PA, Pham TV, Burugu D, Luecke DF (2016). Effect of RpoN, RpoS and LuxS pathways on the biofilm formation and antibiotic sensitivity of *Borrelia Burgdorferi*. Eur. J. Microbiol. Immunol..

[36] Danhorn T, Fuqua C (2007). Biofilm formation by plant-associated bacteria. Annu. Rev. Microbiol..

[37] Rinaudi LV, Giordano W (2010). An integrated view of biofilm formation in rhizobia. FEMS Microbiol. Lett..

[38] Jones K, Bradshaw SB (1996). Biofilm formation by the enterobacteriaceae: A comparison between *Salmonella enteritidis*, *Escherichia coli* and a nitrogen-fixing strain of *Klebsiella pneumoniae*. J. Appl. Bacteriol..

[39] Rinaudi L (2006). Effects of nutritional and environmental conditions on *Sinorhizobium meliloti* biofilm formation. Res. Microbiol..

[40] Meneses CHSG, Rouws LFM, Simoes-Araujo JL, Vidal MS, Baldani JI (2011). Exopolysaccharide production is required for biofilm formation and plant colonization by the nitrogen-fixing endophyte *Gluconacetobacter diazotrophicus*. Mol. Plant Microbe Interact..

[41] Wang D, Xu AM, Elmerich C, Ma LYZ (2017). Biofilm formation enables free-living nitrogen-fixing rhizobacteria to fix nitrogen under aerobic conditions. ISME J..

[42] Pereg-Gerk L, Paquelin A, Gounon P, Kennedy IR, Elmerich C (1998). A transcriptional regulator of the LuxR-UhpA family, FlcA, controls flocculation and wheat root surface colonization by *Azospirillum brasilense* Sp7. Mol. Plant Microbe Interact..

[43] Fujishige NA, Kapadia NN, De Hoff PL, Hirsch AM (2006). Investigations of *Rhizobium* biofilm formation. FEMS Microbiol. Ecol..

[44] Janczarek M (2011). Environmental signals and regulatory pathways that influence exopolysaccharide production in *Rhizobia*. Int. J. Mol. Sci..

[45] Nocelli N, Bogino PC, Banchio E, Giordano W (2016). Roles of extracellular polysaccharides and biofilm formation in heavy metal resistance of *Rhizobia*. Materials.

[46] Ramirez-Mata A, Pacheco MR, Moreno SJ, Xiqui-Vazquez ML, Baca BE (2018). Versatile use of *Azospirillum brasilense* strains tagged with *egfp* and mCherry genes for the visualization of biofilms associated with wheat roots. Microbiol. Res..

[47] Jijon-Moreno S, Baca BE, Castro-Fernandez DC, Ramirez-Mata A (2019). TyrR is involved in the transcriptional regulation of biofilm formation and D-alanine catabolism in *Azospirillum brasilense* Sp7. PLoS ONE.

[48] Yan YL (2008). Nitrogen fixation island and rhizosphere competence traits in the genome of root-associated *Pseudomonas stutzeri* A1501. Proc. Natl Acad. Sci. USA.

[49] Yan YL (2010). Global transcriptional analysis of nitrogen fixation and ammonium repression in root-associated *Pseudomonas stutzeri* A1501. BMC Genomics.

[50] Zhan YH (2016). The novel regulatory ncRNA, NfiS, optimizes nitrogen fixation via base pairing with the nitrogenase gene *nifK* mRNA in *Pseudomonas stutzeri* A1501. Proc. Natl Acad. Sci. USA.

[51] Zhan YH (2019). NfiR, a new regulatory noncoding RNA (ncRNA), is required in concert with the NfiS ncRNA for optimal expression of nitrogenase genes in *Pseudomonas stutzeri* A1501. Appl. Environ. Microbiol..

[52] Zhang HY (2019). The *Pseudomonas stutzeri*-specific regulatory noncoding RNA NfiS targets *katB* mRNA encoding a catalase essential for optimal oxidative resistance and nitrogenase activity. J. Bacteriol..

[53] Ma Y (2016). Identification of the nitrogen-fixing *Pseudomonas stutzeri* major flagellar gene regulator FleQ and its role in biofilm formation and root colonization. J. Integr. Agr..

[54] Dixon R, Kahn D (2004). Genetic regulation of biological nitrogen fixation. Nat. Rev. Microbiol..

[55] Heurlier K, Denervaud V, Pessi G, Reimmann C, Haas D (2003). Negative control of quorum sensing by RpoN (sigma54) in *Pseudomonas aeruginosa* PAO1. J. Bacteriol..

[56] Byrd MS (2009). Genetic and biochemical analyses of the *Pseudomonas aeruginosa* Psl exopolysaccharide reveal overlapping roles for polysaccharide synthesis enzymes in Psl and LPS production. Mol. Microbiol..

[57] Schulmeyer KH (2016). Primary and secondary sequence structure requirements for recognition and discrimination of target RNAs by *Pseudomonas aeruginosa* RsmA and RsmF. J. Bacteriol..

[58] Miller CL (2016). RsmW, *Pseudomonas aeruginosa* small non-coding RsmA-binding RNA upregulated in biofilm versus planktonic growth conditions. BMC Microbiol..

[59] Hindson CM (2013). Absolute quantification by droplet digital PCR versus analog real-time PCR. Nat. Methods.

[60] Kay E, Dubuis C, Haas D (2005). Three small RNAs jointly ensure secondary metabolism and biocontrol in *Pseudomonas fluorescens* CHA0. Proc. Natl Acad. Sci. USA.

[61] Huertas-Rosales O (2017). The *Pseudomonas putida* CsrA/RsmA homologues negatively affect c-di-GMP pools and biofilm formation through the GGDEF/EAL response regulator CfcR. Environ. Microbiol..

[62] Heeb S, Valverde C, Gigot-Bonnefoy C, Haas D (2005). Role of the stress sigma factor RpoS in GacA/RsmA-controlled secondary metabolism and resistance to oxidative stress in *Pseudomonas fluorescens* CHA0. FEMS Microbiol. Lett..

[63] Heredia-Ponce Z, de Vicente A, Cazorla FM, Gutierrez-Barranquero JA (2021). Beyond the wall: exopolysaccharides in the biofilm lifestyle of pathogenic and beneficial plant-associated. Pseudomonas Microorg..

[64] Heredia-Ponce Z (2020). Biological role of EPS from *Pseudomonas syringaepv. syringae* UMAF0158 extracellular matrix, focusing on a Psl-like polysaccharide. npj Biofilms Microbiomes.

[65] Fett WF, Osman SF, Dunn MF (1989). Characterization of exopolysaccharides produced by plant-associated fluorescent pseudomonads. Appl. Environ. Microbiol..

[66] Irie Y (2012). Self-produced exopolysaccharide is a signal that stimulates biofilm formation in *Pseudomonas aeruginosa*. Proc. Natl Acad. Sci. USA.

[67] Tseng BS (2013). The extracellular matrix protects *Pseudomonas aeruginosa* biofilms by limiting the penetration of tobramycin. Environ. Microbiol..

[68] Postgate J (1989). Trends and perspectives in nitrogen fixation research. Adv. Microb. Physiol..

[69] Schafer A (1994). Small mobilizable multi-purpose cloning vectors derived from the *Escherichia coli* plasmids pK18 and pK19: selection of defined deletions in the chromosome of *Corynebacterium glutamicum*. Gene.

[70] Figurski DH, Helinski DR (1979). Replication of an origin-containing derivative of plasmid RK2 dependent on a plasmid function provided in trans. Proc. Natl Acad. Sci. USA.

[71] Desnoues N (2003). Nitrogen fixation genetics and regulation in a *Pseudomonas stutzeri* strain associated with rice. Microbiol. SGM.

[72] Jerabek-Willemsen M, Wienken CJ, Braun D, Baaske P, Duhr S (2011). Molecular interaction studies using microscale thermophoresis. Assay. Drug Dev. Technol..

[73] Wang Y, Cen XF, Zhao GP, Wang J (2012). Characterization of a new GlnR binding box in the promoter of *amtB* in *Streptomyces coelicolor* inferred a PhoP/GlnR competitive binding mechanism for transcriptional regulation of *amtB*. J. Bacteriol..

[74] Spangler C, Bohm A, Jenal U, Seifert R, Kaever V (2010). A liquid chromatography-coupled tandem mass spectrometry method for quantitation of cyclic di-guanosine monophosphate. J. Microbiol. Methods.

