# Supplementary material for: A regulatory network involving Rpo, Gac and Rsm for nitrogen-fixing biofilm formation by Pseudomonas stutzeri
Source: NPJ Biofilms Microbiomes. 2021 Jul 1;7:54. doi: 10.1038/s41522-021-00230-7 (PMC8249394; doi:10.1038/s41522-021-00230-7)
Supplement: Supplementary file 1 — Supplementary Information [file 41522_2021_230_MOESM1_ESM.pdf]

Supplementary information

Supplementary Figures

Supplementary Figure 1

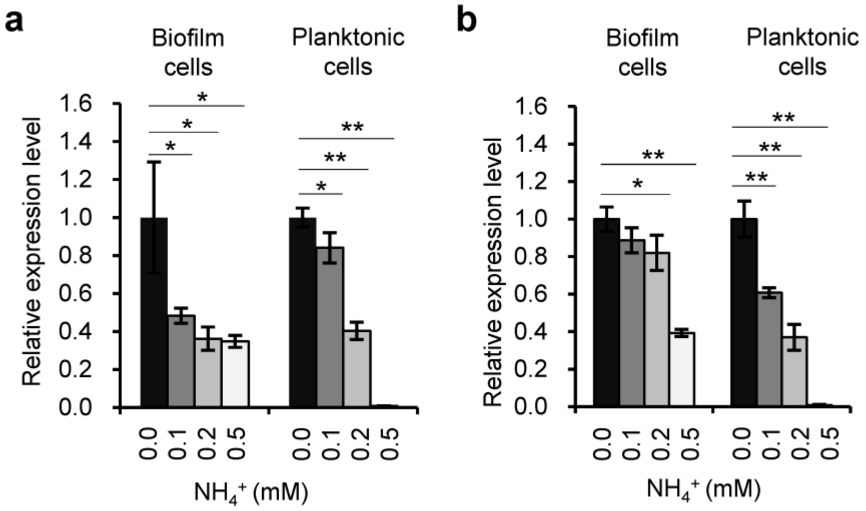

Supplementary Figure 1 Effect of NH_4^+ concentration on the expression of the *nifA* (a) and *nifH* (b) genes in the planktonic and mature biofilm cells under the same conditions as in Fig. 1f. Each error bar indicates the standard deviation of three independent experiments. Asterisks indicate statistical significance by one-way ANOVA with LSD multiple-comparison test: *, $p<0.05$; **, $p<0.01$.

Supplementary Figure 2

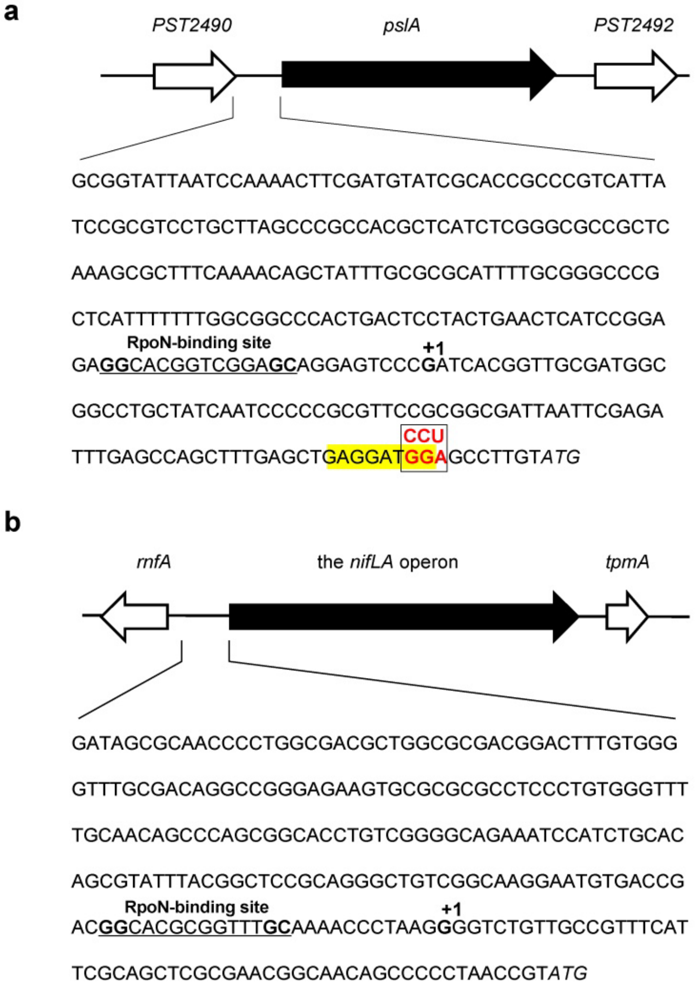

Supplementary Figure 2 Nucleotide sequences of the *pslA* (a) and *nifLA* (b) promoter regions. The RpoN binding sites are underlined. +1 indicates the transcription start site based on the 5' RACE analysis. The RsmA binding site with GGA elements in the *pslA* 5'-UTR region, as previously reported (Dubey *et al*., 2005), is shown in red. The GGA motif is shown in red, and the corresponding mutated sequence in the synthesized oligonucleotide N-PslA-mut, containing the 5'-UTR of *pslA* mRNA, is shown above the GGA motif. The putative ribosome binding (RBS) site is highlighted in yellow.

Supplementary Figure 3

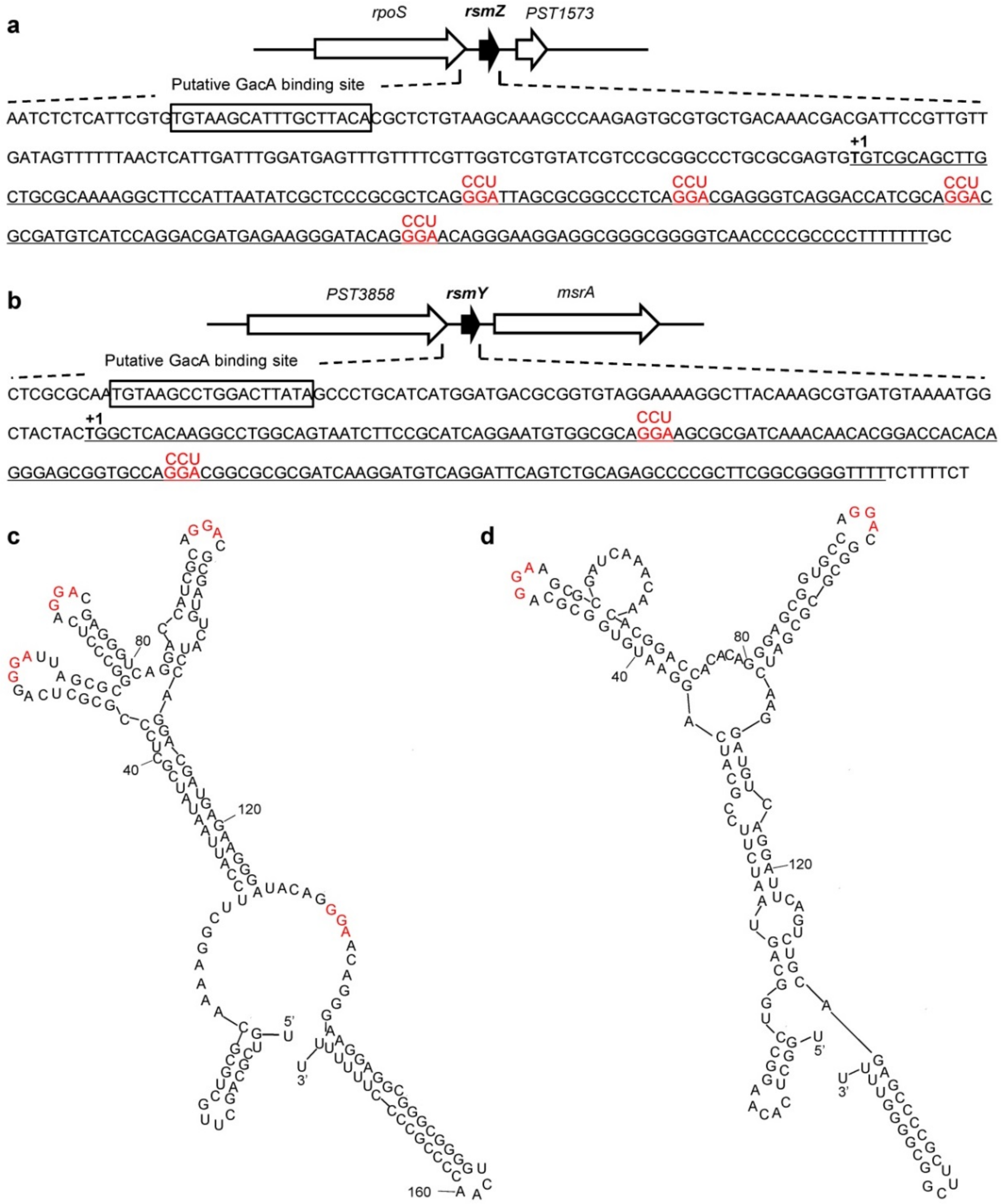

Supplementary Figure 3 ***In silico*** **analyses to document the involvement of RsmZ and RsmY in RsmA regulation. a**, **b**, RsmZ and RsmY nucleotide sequences: +1 indicates transcription start site based on a 5’ RACE analysis. The palindromic sequence (TGTAAG-N6-CTTACA, where N is any nucleotide), boxed from -83 to -66 for *rsmY* and from -174 to -191 for *rsmZ*, denotes the upstream activation sequence (UAS), a potential GacA regulatory site that is highly conserved in GacA-regulated genes (Kay *et al*., 2005). The nucleotide sequences of *rsmY* and *rsmZ* are underlined. GGA motifs predicted to be located on the single-stranded outer stem loops are shown in red, and the corresponding mutated sequences in the oligonucleotides (N-RsmZ-mut and N-RsmY-mut) for further MST analysis are shown above the GGA motif. **c**, **d**, Predicted secondary structures of RsmZ and RsmY obtained at 30 °C using the mfold web server (http://unafold.rna.albany.edu/?q=mfold). Several GGA motifs were predicted to be located on the single-stranded outer stem loops, which are patterns reminiscent of that of the Rsm ncRNAs, thus suggesting a role in binding/sequestering RsmA.

Supplementary Figure 4

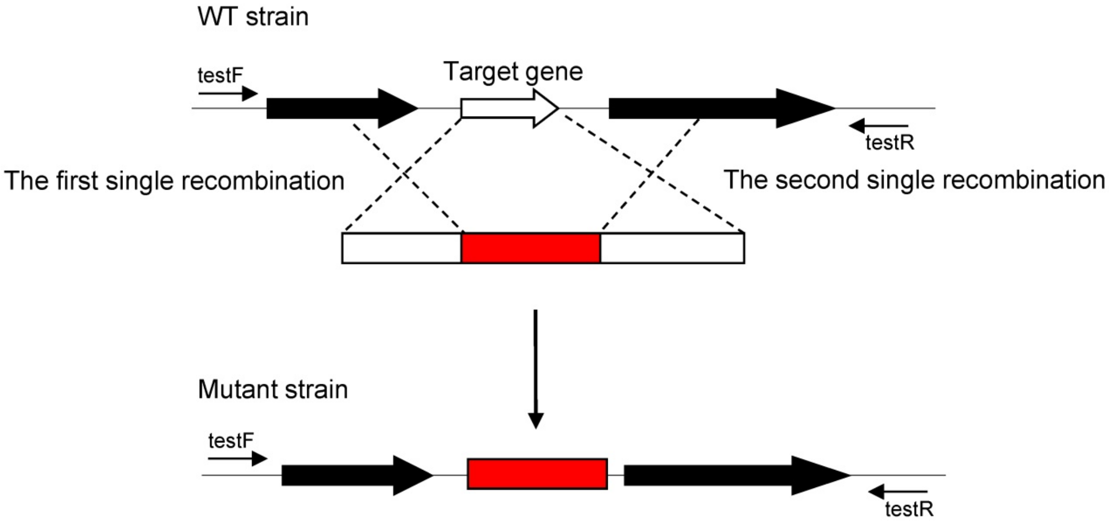

Supplementary Figure 4 Schematic representation of deletion mutant construction by homologous suicide plasmid integration. Primers testF and testR, used for analysis of the target gene deletion, are shown by arrows. The red box represents the resistance gene.

Supplementary Table 1 Strains and plasmids used in this study

Strain/plasmid	Relevant characteristics	Source/reference
**Strain**		
A1501	WT, Chinese culture collection: CGMCC 0351	
ATCC17588	WT, Chinese Culture Collection: CGMCC 1.1803	
Ab1622, Δ*gacA*	*gacA* deletion mutant, Cm^R	This study
Ab1622 (pL*gacA*)	*gacA* deletion mutant containing pL*gacA*, Tc^R and Cm^R	This study
Ab1632, Δ*rsmA*	*rsmA* insertion mutant, Km^R	This study
Ab1632 (pL*rsmA*)	*rsmA* mutant containing pL*gacA*, Tc^R and Km^R	This study
A1501 (pL*rsmA*)	A1501 containing pL*rsmA*, Tc^R	This study
Ab1642, Δ*rsmZ*	*rsmZ* deletion mutant, Cm^R	This study
Ab1642 (pL*rsmZ*)	*rsmZ* deletion mutant containing pL*rsmZ*, Tc^R and Cm^R	This study
A1501 (pL*rsmZ*)	A1501 containing pL*rsmZ*, Tc^R	This study
Ab1643, Δ*rsmY*	*rsmY* deletion mutant, Gm^R	This study
Ab1643 (pL*rsmY*)	*rsmY* deletion mutant containing pL*rsmY*, Tc^R and Gm^R	This study
A1501 (pL*rsmY*)	A1501 containing pL*rsmY*, $Tc^{R.}$	This study
Ab1644, Δ*rsmZY*	*rsmY* and *rsmZ* double deletion mutants, Gm^R and Cm^R	This study
Ab1652, Δ*bifA*	*bifA* insertion mutant, Km^R	This study
Ab1652 (pL*bifA*)	*bifA* mutant containing pL*bifA,* Tc^R and Km^R	This study
A1501 (pL*bifA*)	A1501 containing pL*bifA*, Tc^R	This study
Ab1662, Δ*sadC*	*sadC* deletion mutant, Gm^R	This study
Ab1662 (pL*sadC*)	*sadC* deletion mutant containing pL*sadC,* Tc^R and Gm^R	This study
A1501 (pL*sadC*)	A1501 containing pL*sadC*, Tc^R	This study
A1550, Δ*rpoN*	*rpoN* deletion mutant, Cm^R	Desnoues N, *et al.* 2003
A1550 (pL*rpoN*)	*rpoN* deletion mutant containing pL*rpoN,* Tc^R and Cm^R	This study

Δ*pslA*	*pslA* insertion mutant, Km^R	This study
Δ*pslA* (pL*pslA*)	*pslA* insertion mutant containing pL*pslA,* c^R and Km^R	This study
A1507, Δ*rpoS*	*rpoS* deletion mutant	Zhan YH, *et al.* 2016
A1507 (pL*rpoS*)	*rpoS* deletion mutant containing pL*rpoS,* Tc^R	This study
A1506, Δ*nifA*	*nifA* deletion mutant, Cm^R	Desnoues N, *et al.* 2003
A1506 (pL*nifA*)	*nifA* deletion mutant containing pL*nifA,* Tc^R and Cm^R	Desnoues N, *et al.*2003
**Plasmid**		
pK18*mob*	Mobilizable plasmid containing an *E. coli* origin of replication, Km^R	Lab collection
pK18*mobsacB*	Suicide plasmid for gene deletion mutation, Km^R	Lab collection
pLAFR3	For complement strain construction, Tra$^-$, mob$^+$, cos, RK2 replicon, Tc^R	Lab collection
pRK2013	Helper plasmid for conjugation, Km^R	Lab collection
pKatCAT5	Source of Cm resistance cassette, Cm^R	Lab collection
pJN105	Source of Gm resistance cassette, Cm^R	Lab collection
pGEM-T Easy	Cloning vector, Amp^R	Promega
pTWIN1	Plasmid for expression of recombinant target protein with anti-chitin binding domain	Lab collection
pET28a	Plasmid for expression of recombinant target protein with 6xHis-tag.	Lab collection
pTWIN1-RsmA	pTWIN1 containing the *rsmA* gene for RsmA expression and purification	This study
pET28a-RpoN	pET28a derivative containing the *rpoN* gene for RpoN expression and purification	This study
pL*gacA*	pLAFR3 derivative carrying the *gacA* gene under the control of its endogenous promoter, Tc^R	This study
pL*rsmA*	pLAFR3 derivative carrying the *rsmA* gene under the control of its endogenous promoter, Tc^R	This study
pL*rsmZ*	pLAFR3 derivative carrying the *rsmZ* gene under the control of its endogenous promoter, Tc^R	This study
pL*rsmY*	pLAFR3 derivative carrying the *rsmY* gene under the control of its endogenous promoter, Tc^R	This study
pL*sadC*	pLAFR3 derivative carrying the *sadC* gene under the control of its endogenous promoter, Tc^R	This study
pL*bifA*	pLAFR3 derivative carrying the *bifA* gene under the control of its endogenous promoter, Tc^R	This study
pL*rpoN*	pLAFR3 derivative carrying the *rpoN* gene under the control of its endogenous promoter, Tc^R	This study

pL*pslA*	pLAFR3 derivative carrying the *pslA* gene under the control of its endogenous promoter, Tc^R	This study
pL*rpoS*	pLAFR3 derivative carrying the *rpoS* gene under the control of its endogenous promoter, Tc^R	This study
pL*nifA*	pLAFR3 derivative carrying the *nifA* gene under the control of its endogenous promoter, Tc^R	This study

Supplementary Table 2 Primers used in this study

Primer*	Sequence (5' – 3')▲	purpose
P18con-F	GCCGATTCATTAATGCAGCTGGCAC	Test for insertion mutant construction
M-*gacA*(up)-F	GCGGATCCGATTTGATCAGCCGCTCCAGCCCGT	*gacA* deletion mutant construction
M-*gacA*(up)-R	AAGGGCCCTCGGTCTCCATGCCAGCACCCTAATCAAGCAGACACC	
M-*gacA*(CmR)-F	TGTCTGCTTGATTAGGGTGCTGGCATGGAGACCGAGGGCCCTTGA	
M-*gacA*(CmR)-R	GAGGAATCGAAGGCTTCTGTCATATTACGCCCCGCCCTGCCACTCAT	
M-*gacA*(down)-F	ATGAGTGGCAGGGCGGGGCGTAATATGACAGAAGCCTTCGATTCCTCG	
M-*gacA*(down)-R	GGAAGCTTGCTGTCCTCGCATTGCCGCACGAAG	
C-*gacA*-F	CCATGATTACGAATTCCCGGGGATCCGATTTGATCAGCCGCTCCAGCC	*gacA* complement plasmid construction
C-*gacA*-R	GTAAAACGACGGCCAGTGCCAAGCTTGAACATGCGATAGACGCCCGGC	
M-*rsmA*-F	CCGGAATTCGATTCTGACTCGCCGGGTAG	*rsmA* insertion mutant construction
M-*rsmA*-R	CGCGGATCCGGCTTGGTTCCTGATCCT	
C-*rsmA*-F	CGGAATTCCAAAGTTTCGGTTGTCATTGAAGAG	*rsmA* complement plasmid construction
C-*rsmA*-R	CCAAGCTT TTAGTGGCTTGGTTCCTGATCCTTC	
M-*rsmZ*(up)-F	CCGGATCCCGTCTGCGCGAAATACTGGAGCGA	*rsmZ* deletion mutant construction
M-*rsmZ*(up)-R	ACAGTACTGCGATGAGTGGCAGGGCGGGAGCGATATTAATGGAAGCCT	
M-*rsmZ*(CmR)-F	AGGCTTCCATTAATATCGCTCCCGCCCTGCCACTCATCGCAGTACTGT	
M-*rsmZ*(CmR)-R	GGTTCAGCTGGAGTTTCCGGCGGGCATGGAGACCGAGGGCCCTTGACA	
M-*rsmZ*(down)-F	TGTCAAGGGCCCTCGGTCTCCATGCCCGCCGGAAACTCCAGCTGAACC	
M-*rsmZ*(down)-R	CCAAGCTTACGTGTTCATGGCGCGGCTGCCTCG	
C-*rsmZ*-F	CGGAATTCCGTCTGCGCGAAATACTGGAGCGA	*rsmZ* complement plasmid construction
C-*rsmZ*-R	CCAAGCTT GGTTCAGCTGGAGTTTCCGGCGGG	
M-*rsmY*(up)-F	GCGGATCCGCGGTGGGCTACGCCGGAGGGATTA	*rsmY* deletion mutant construction
M-*rsmY*(up)-R	ACAAGGTGCTGATGCCGCTGGCGATTTCTTTTCTGCCAAGTCCGCCGTTT	
M-*rsmY*(GmR)-F	AAACGGCGGACTTGGCAGAAAAGAAATCGCCAGCGGCATCAGCACCTTGT	
M-*rsmY*(GmR)-R	GCAGTAATCTTCCGCATCAGGAATGTTAGGTGGCGGTACTTGGGTCGATA	
M- *rsmY*	TATCGACCCAAGTACCGCCACCTAACATTCCTGATGCGG	

(down)-F	AAGATTACTGC	
M-*rsmY*(down)-R	GGAAGCTTAGCTGACCGCCTGGGTGATCGATCA	
C-*rsmY*-F	CCCAAGCTTGGATTACGCCGAACCCGACC	*rsmY* complement plasmid construction
C-*rsmY*-R	CCGGAATTCTTAATCTAAAGGCAATATGA	
M-*algU*-F	GGAAACAGCTATGACCATGATTACGAATTCATGCTGACTCAGGAGCAGGACCAGC	*algU* insertion mutant construction
M-*algU*-R	GCATGCCTGCAGGTCGACTCTAGAGGATCCGCGACGTCCTGAGCTTCGTGGGAG	
C-*algU*-F	CGGAATTCAACTCCGCCCTGAGCCCAATAGGT	*algU* complement plasmid construction
C-*algU*-R	CCAAGCTT TTCCTCTCTTGGCTGTCGCCGCTGTC	
M-*sadC*(up)-F	CGTTGTAAAACGACGGCCAGTGCCAGCGCGGCGTACGCCCTCGTTGATCA	*sadC* deletion mutant construction
M-*sadC*(up)-R	CTTCATCCGTTTCCACGGTGTGCGTAGGGATCCATCCCACTGTGGCAATT	
M-*sadC*(GmR)-F	AATTGCCACAGTGGGATGGATCCCTACGCACACCGTGGAAACGGATGAAG	
M-*sadC*(GmR)-R	CCAACGACGTCCGGCGCAGCGGAGTTTAGGTGGCGGTACTTGGGTCGATA	
M-*sadC*(down)-F	TATCGACCCAAGTACCGCCACCTAAACTCCGCTGCGCCGGACGTCGTTGG	
M-*sadC*(down)-R	TACGAATTCGAGCTCGGTACCCGGGGACGCAATTCACGCGCGAAGTCGCA	
C-*sadC*-F	CGGAATTCGCGCGGCGTACGCCCTCGTTGATCA	*sadC* complement plasmid construction
C-*sadC*-R	CCAAGCTT CCAACGACGTCCGGCGCAGCGGAGT	
M-*bcsA*(up)-F	GCGGATCCGCACCTGCTGCCGTACGGCGAGCT	*bcsA* deletion mutant construction
M-*bcsA*(up)-R	ATGTCAAGGGCCCTCGGTCTCCATGATCAGCACTTCGCTCGCTGGGCCAG	
M-*bcsA*(CmR)-F	CTGGCCCAGCGAGCGAAGTGCTGATCATGGAGACCGAGGGCCCTTGACAT	
M-*bcsA*(CmR)-R	AGCGTCCGGGAATAGGTTCGGGTGAATTACGCCCCGCCCTGCCACTCATC	
M-*bcsA*(down)-F	GATGAGTGGCAGGGCGGGGCGTAATTCACCCGAACCTATTCCCGGACGCT	
M-*bcsA*(down)-R	GGAAGCTTTCGTCAGAGCCGGGTCAGCCTGCAT	
C-*bcsA*-F	ACACAGGAAACAGCTATGACCATGATTACGGGATGGACGCCCCTCACGGCACGCA	*bcsA* complement plasmid construction
C-*bcsA*-R	GCCAGTGCCAAGCTTGGCTGCAGGTCGACGTCATGACGGGGTTACCAGGGATTTA	
M-*pslA*-F	AGGAAACAGCTATGACATGATTACGATGCGCGTACAGTCTGTTGGAACCC	*pslA* insertion mutant construction
M-*pslA*-R	GCATGCCTGCAGGTCGACTCTAGAGTGGAAGATGATGACGCTGAGTACCG	

C-*pslA*-F	ACACAGGAAACAGCTATGACCATGATTACGGGCTCCCAGGTGACACGAACCGCCA	*pslA* complement plasmid construction
C-*pslA*-R	GCCAGTGCCAAGCTTGGCTGCAGGTCGACGTCAGTACACGTCCTTGTTCAGCAGC	
RT-*16S*-F	CCTACGGGAGGCAGCAG	qRT-PCR
RT-*16S*-R	ATTACCGCGGCTGCTGG	
RT-*gacS*-F	GACCAGATGCAGAGCACCAT	
RT-*gacS*-R	GTAGTCGGGCAGAGAACCAC	
RT-*gacA*-F	GAAGAGATGGTGCAGGCCA	
RT-*gacA*-R	TTTCCCGTTCGGAAAGCAGA	
RT-*rsmA*-F	GTGGGTGACGATGTGACTGT	
RT-*rsmA*-R	GTGGCTTGGTTCCTGATCCT	
RT-*rsmZ*-F	CTCAGGACGAGGGTCAGGA	
RT-*rsmZ*-R	TCCCTGTTCCCTGTATCCCTT	
RT-*rsmY*-F	GCGATCAAACAACACGGACC	
RT-*rsmY*-R	GGGCTCTGCAGACTGAATCC	
RT-*algU*-F	TGCGTACGGCTCTAACACTG	
RT-*algU*-R	TATCTATGGCTTCACGCGCC	
RT-*sadC*-F	TGGCGAGTGGTTTCTACGAG	
RT-*sadC*-R	ATAGAGCACGAGCAGTGAGC	
RT-*bifA*-F	GAAGGCGTCGAAACTCCTGA	
RT-*bifA*-R	GGCAGGGGCTTGCTGTAATA	
RT-*bcsA*-F	TCCAGGAGTAGGTCTCTGCC	
RT-*bcsA*-R	TCGTGCTGTCAGCCATCATT	
RT-*pslA*-F	CTCACCCGAGTTTCGTCGAT	
RT-*pslA*-R	ATGATGACGCTGAGTACCGC	
RT-*rpoN*-F	CTTCTTCTCCAGCCACGTCAG	
RT-*rpoN*-R	CCAGTAAACCAGCGATCTTGC	
RT-*rpoS*-F	GCATGATCGAAAGCAACCTG	
RT-*rpoS*-R	CAGATAGACATTCAGCTCCTTCAC	
RT-*pilA*-F	TGTTGCAAGTAACGGAGCCT	
RT-*pilA*-R	TTTAGTGTTGCGGAGAGCGT	
RT-*fleR*-F	GACCAGATGCAGAGCACCAT	
RT-*fleR*-R	GTAGTCGGGCAGAGAACCAC	
RT-*cheA*-F	GACCGAGATTTCCGACGTGT	
RT-*cheA*-R	TTGATCACCACCTTCGAGCC	
RT-*nifA*-F	CGCGAAGACCTCTACTACCG	
RT-*nifA*-R	CAGCTTGAGTTTGCGACCCT	
RT-*nifH*-F	GAGATGATGGCGATGTATGC	
RT-*nifH*-R	GGTCGGTGTTGCGGCTGTTG	
rsmZ-GSP1	TCCCTGTTCCCTGTATCCCT	5' RACE for *rsmZ*
rsmZ-GSP2	GTCCTGGATGACATCGCGTCCTGC	
rsmY-GSP1	GCTCTGCAGACTGAATCCTGACAT	5' RACE for *rsmY*

rsmY-GSP2	ACATCCTTGATCGCGCGCCGTCCT	
rpoS-GSP1	CGACAGATCGTCATCCAGCA	5’ RACE for *rpoS*
rpoS-GSP2	TGGTCGATCGTCAGTAAGCG	
rpoS-GSP3	GGTCGCGTAGGTAGAGAAGC	
pslA-GSP1	CACCTGGGTGACCTTTTCCT	5’ RACE for *pslA*
pslA-GSP2	CAAGGATCACTGCGTTCTGC	
pslA-GSP3	ACCAGGTGTTTGGGTTCCAG	
nifL-GSP1	TGCCGTCGAGCAGCTCTTCC	5’ RACE for *nifL*
nifL-GSP2	TTGTGCTGGTCGCTGCTGT	
nifL-GSP3	ATGGTCCGCTCGTGCTCGTC	
pTWIN1-*rsmA*-F	GGCATATGCTGATTCTGACTCGCCGGGTAGG	RsmA protein expression vector construction
pTWIN1-*rsmA*-R	GGGAATTCGTGGCTTGGTTCCTGATCCTTCT	
pET28a-*rpoN*-F	GCGGCCTGGTGCCGCGCGGCAGCCATATGATGAAACCATCGCTAGTCCTGAAG	RpoN protein expression vector construction
pET28a-*rpoN*-R	TGGTGGTGCTCGAGTGCGGCCGCAAGCTTTCACAGCAATCGTTTACGCTCGC	
RsmZ-probe-R(DIG)	TCCCTGTTCCCTGTATCCCTT	Northern blot for *rsmZ*
RsmY-probe-R(DIG)	GGGCTCTGCAGACTGAATCC	Northern blot for *rsmY*
FP-*nifA*-F	GGACTTTGTGGGGTTTGCG	Footprinting assay
FP-*nifA*-R	GTCATGTGCATGTCCTCATCG	
FP-*pslA*-F	TATCGCACCGCCCGTCAT	
FP-*pslA*-R	GCATACAAGGCTCCATCCTCA	

*F, Forward; R, Reverse

▲Restriction sites are underlined.
